# In Vivo Biocatalytic Cascades Featuring an Artificial‐Enzyme‐Catalysed New‐to‐Nature Reaction**

**DOI:** 10.1002/anie.202214191

**Published:** 2022-11-30

**Authors:** Linda Ofori Atta, Zhi Zhou, Gerard Roelfes

**Affiliations:** ^1^ Stratingh Institute for Chemistry University of Groningen Nijenborgh 4 9747 AG Groningen The Netherlands; ^2^ Current address: School of Life Science and Health Engineering Jiangnan University Wuxi 214122 China

**Keywords:** Artificial Enzymes, Biocatalysis, Biosynthetic Pathways, Hydrazone Formation, In Vivo Cascades

## Abstract

Artificial enzymes utilizing the genetically encoded non‐proteinogenic amino acid *p‐*aminophenylalanine (pAF) as a catalytic residue are able to react with carbonyl compounds through an iminium ion mechanism to promote reactions that have no equivalent in nature. Herein, we report an in vivo biocatalytic cascade that is augmented with such an artificial enzyme‐catalysed new‐to‐nature reaction. The artificial enzyme in this study is a pAF‐containing evolved variant of the lactococcal multidrug‐resistance regulator, designated LmrR_V15pAF_RMH, which efficiently converts benzaldehyde derivatives produced in vivo into the corresponding hydrazone products inside *E. coli* cells. These in vivo biocatalytic cascades comprising an artificial‐enzyme‐catalysed reaction are an important step towards achieving a hybrid metabolism.

## Graphical abstract

We report in vivo biocatalytic cascade reactions comprising a combination of canonical enzyme‐catalysed reactions with an artificial‐enzyme‐catalysed new‐to‐nature reaction. The artificial enzyme contains a genetically encoded unnatural catalytic residue, which catalyses the formation of a hydrazone product from biosynthetically produced benzaldehydes in *E. coli*.

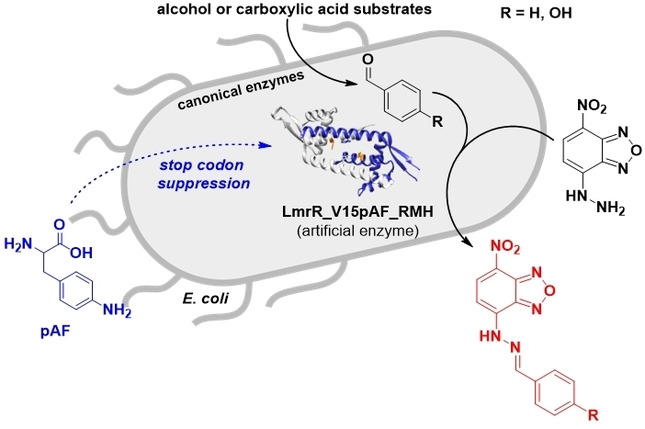

## Introduction

Cellular metabolism uniquely demonstrates the power of biocatalytic cascade reactions in living cells. Molecular structures of remarkable complexity are produced through an intricate network of interconnected biocatalytic reactions. Such biological systems have proven amenable to modification by metabolic engineering, affording modified products.^[1]^ However, the structural diversity that can be achieved is inherently limited by the synthetic repertoire available to nature. In contrast, chemical synthesis offers virtually unlimited versatility in reaction scope; however, to date, synthetic chemical systems cannot rival the sophistication of nature. Hence, an attractive solution would be to combine the best of both worlds to create a “hybrid metabolism”, which augments biological synthesis with abiological catalytic chemistry.^[2]^

Most efforts towards this goal have, so far, focused on supplementing biological synthesis with a transition‐metal‐ or organocatalysed reaction.[^3^, ^4^, ^5^, ^6^, ^7^, ^8^, ^9^, ^10^] While some encouraging results have been obtained, the low activity of transition‐metal complexes and organocatalysts in biological systems, as well as their potential mutual incompatibility with biocatalysts, is often a limiting factor. An alternative approach involves using artificial enzymes created by integrating a synthetic catalyst into a protein scaffold.^[11]^ The protein environment helps to accelerate the reaction by providing additional interactions, but also protects the chemical catalyst from the cellular environment.^[2]^ In the last decade, the first examples of the application of artificial metalloenzymes in living cells were reported.[^12^, ^13^, ^14^, ^15^, ^16^, ^17^, ^18^, ^19^] Biocatalytic cascades involving artificial metalloenzymes have also been reported, although these were carried out in vitro, using isolated proteins.[^20^, ^21^, ^22^] However, in one recent example, an iridium‐porphyrin‐substituted cytochrome P450 monooxygenase was employed to catalyse cyclopropanation of a biosynthesised terpene in a heavily engineered *Escherichia coli* strain.^[23]^

Previously, we introduced the concept of using genetically encoded non‐canonical amino acids as organocatalytic residues in artificial enzymes.[^24^, ^25^] Herein, we now report the application of an artificial enzyme containing a catalytic non‐canonical *p‐*aminophenylalanine (pAF) residue in *E. coli* and its integration into in vivo biocatalytic cascades. The non‐canonical amino acid pAF was incorporated into the lactococcal multidrug‐resistance regulator (LmrR)^[26]^ using the amber stop codon suppression methodology.[^24^, ^27^, ^28^] pAF contains an aniline side chain that can react with aldehydes to form transient iminium ion species that are versatile reactive intermediates in organocatalysis.^[29]^ LmrR with pAF at position 15, designated LmrR_V15pAF, was shown to catalyse the abiotic reaction of benzaldehyde derivatives with hydrazines to form hydrazones.[^24^, ^30^, ^31^] Subsequent directed evolution gave rise to the variant with mutations A92R_N19 M_F93H, designated LmrR_V15pAF_RMH, which showed a 57‐fold increase in catalytic efficiency compared to the parent LmrR_V15pAF.^[32]^ LmrR_V15pAF and evolved variants have also been used successfully in conjugate addition reactions to enals, exploiting the same iminium ion activation strategy.[^33^, ^34^]

**Scheme 1:**
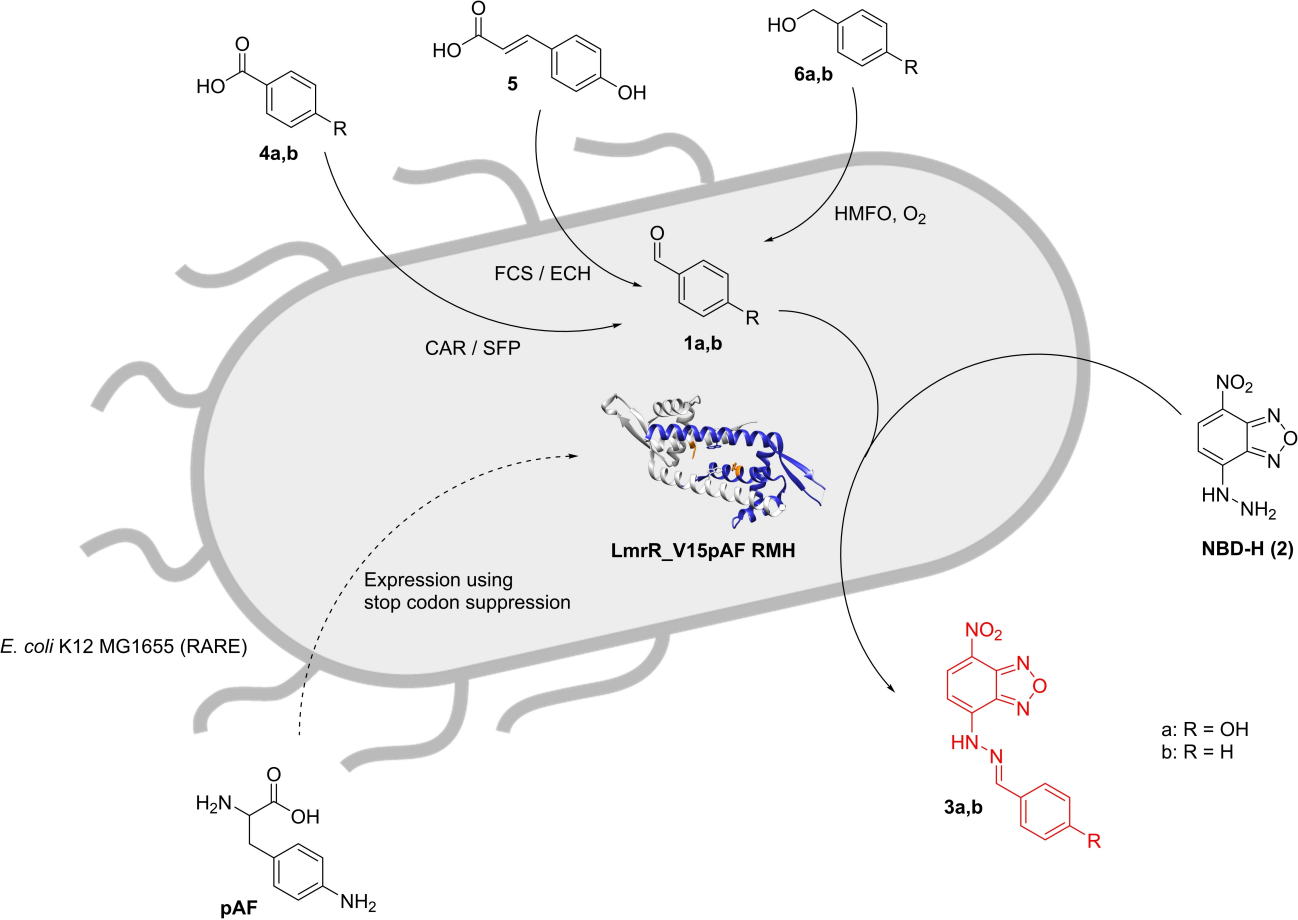
In vivo biocatalytic cascade featuring an abiological hydrazone formation reaction catalysed by the designer enzyme LmrR_V15pAF_RMH.

Since LmrR_pAF is an artificial enzyme that is fully genetically encoded and thus can be biosynthesized in living cells, we surmised that the catalysed hydrazone formation reaction could also be carried out in vivo. Moreover, since a variety of enzymatic syntheses of aldehydes from carboxylic acid or alcohol precursors are known,[^35^, ^36^, ^37^] we envisioned in vivo biocatalytic cascades comprising the biosynthesis of aldehydes using canonical enzymes followed by the LmrR_V15pAF_RMH catalysed new‐to‐nature hydrazone formation reaction (Scheme 1). This represents a step towards a hybrid metabolism, in which artificial‐enzyme‐catalysed abiological reactions are combined with canonical‐enzyme‐catalysed reactions.

## Results and Discussion

In our earlier work, it proved most efficient to introduce pAF indirectly, by first introducing *p‐*azidophenylalanine (pAzF) followed by Staudinger reduction with tris(2‐carboxyethyl)phosphine (TCEP), during the purification process.^[24]^ Direct incorporation of pAF by amber stop codon suppression, using the dedicated orthogonal translation system (OTS) (pDULE2‐para‐aminoPhe),^[28]^ was possible, but proved to give significant misincorporation of other amino acids under standard expression conditions. However, for applications in vivo, indirect incorporation via pAzF cannot be used, since the Staudinger reduction cannot be carried out in vivo. Hence, our initial efforts focused on improving the direct incorporation of pAF by optimizing the expression conditions. For this purpose, we tested the expression of the evolved variant of LmrR_V15pAF containing three mutations, N19M_A92R_F93H (LmrR_V15pAF_RMH).^[32]^ Additionally, the protein includes two mutations in the DNA binding domain, D55 K_Q59 K, and contains a C‐terminal Strep*‐*tag. Three different media (Lysogeny Broth (LB), M9 minimal medium and minimal medium with vitamins (MMV)) were evaluated for protein expression under different conditions. In all cases, overexpression of full‐length protein was observed. MS analysis of the isolated proteins showed a mixture of the desired LmrR_V15pAF_RMH and variants resulting from mis‐incorporation of, in particular, phenylalanine at position 15, albeit with varying ratios depending on the conditions used. The relative intensities of the peaks suggested that expression in MMV gave rise to the largest fraction of pAF incorporation (Figure S6). This is most likely due to the fact that in MMV medium phenylalanine is only available through biosynthesis, and thus present in low concentrations, limiting the potential for misincorporation by the OTS. In the absence of added pAF, misincorporation by phenylalanine was observed as the main species (Figure S9).

As a more quantitative measure for incorporation of pAF, the kinetics of the catalysed hydrazone formation reaction between *p‐*hydroxybenzaldehyde (**1 a**) and 4‐hydrazino‐7‐nitro‐2,1,3‐benzoxadiazole (NBD−H, **2**) were determined and compared to those of LmrR_V15pAF_RMH prepared independently through indirect incorporation.^[32]^ The protein produced in LB and M9 media only showed low activity, suggesting that in these cases mostly misincorporation of other amino acids occurred, consistent with the MS results. In contrast, the protein expressed in MMV medium at 24 °C for 48 h gave good activity. Based on the comparison of the catalytic efficiency it was concluded that the incorporation efficiency of pAF was ≈80 % (Table S2). This was deemed sufficient for in vivo catalysis experiments. Further support for the presence of pAF was obtained in a reductive amination experiment with benzaldehyde and NaCNBH_3_. As expected, a mass increase of 90 was observed, consistent with addition of a benzyl fragment, whereas this was not observed in case of the corresponding LmrR_V15Y variant (Figure S20). This experiment further supports the presence of the pAF and argues against significant misincorporation of Y at this position. Additionally, the incorporation of pAF at position 15 was confirmed by trypsin digest (Figure S5).

**Figure 1:**
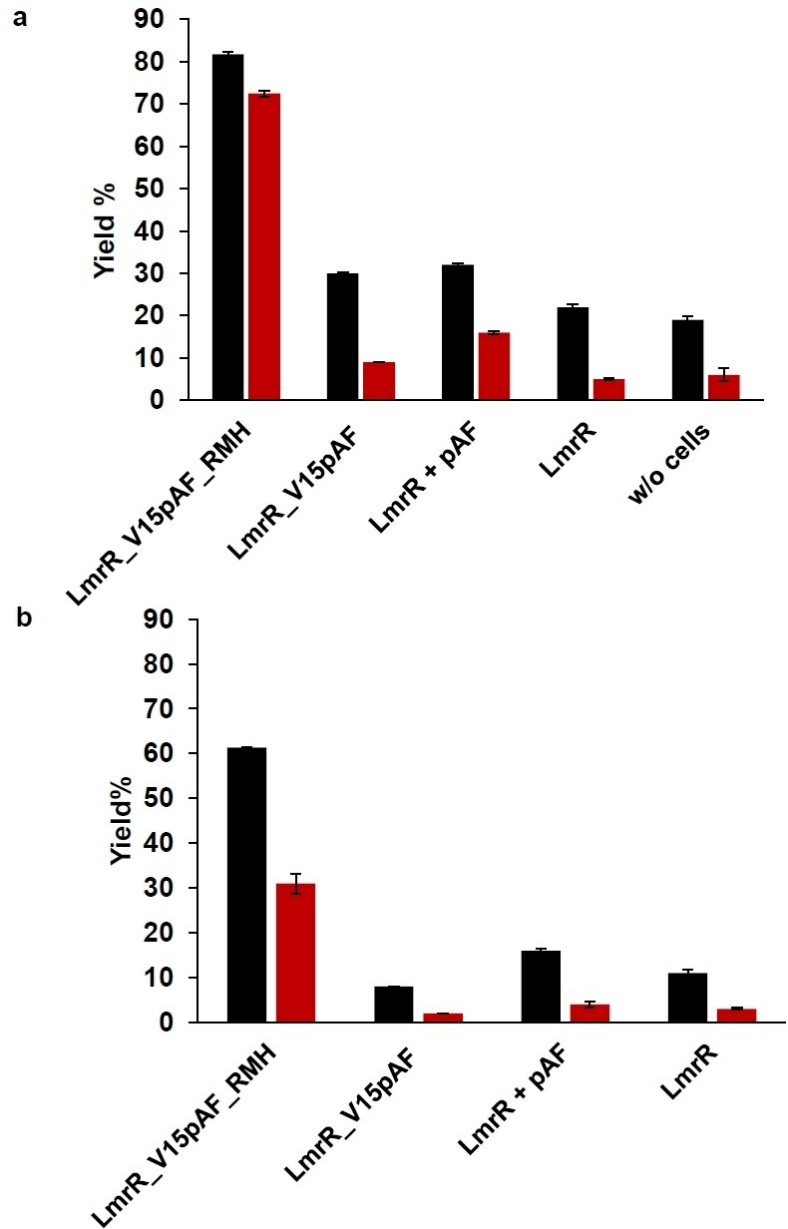
Yield of hydrazone products using MMV medium (black) and phosphate buffer (red) at pH 7 in whole cell (*E.coli* K12 MG1655 (RARE)) catalysed reactions. Conditions: 5 mM **1 a/b** and 50 μM **2** at 24 °C/135 rpm for 3 h. a) Yield of **3 b**; b) yield of **3 a**. Error margins represent standard deviations from experiments performed in triplicate.

The activity of whole *E. coli* cells expressing either wild‐type LmrR, that is, without pAF, LmrR_V15pAF or LmrR_V15pAF_RMH in the hydrazone formation reaction was tested with exogenously provided aldehydes and NBD−H. First, a set of LmrR variants were tested for the hydrazone formation from both *p‐*hydroxybenzaldehyde (**1 a**) and benzaldehyde (**1 b**) in MMV and phosphate buffer pH 7 for 3 h with *E. coli* K‐12 MG1655 (DE3) cells. This strain allows for aromatic aldehyde accumulation because it has a reduced aromatic aldehyde reduction (RARE) activity,^[38]^ as it has been engineered to reduce the reduction of aldehydes by endogenous ketoreductases and alcohol dehydrogenases. Control experiments without cells or with wild type LmrR gave up to about 20 % yield of the corresponding hydrazone products **3 a** and **3 b**, respectively, in MMV medium, while only a low yield was observed in buffer (Figure 1). This shows that there is some background reaction in MMV medium, which is most likely due to the presence of various aromatic amines that can also catalyse the reaction to some extent. Indeed, when increasing the concentration of the most likely culprits, *p‐*aminobenzoic acid, folic acid and thiamine, an increased yield of hydrazone product was observed (Figure S16). This confirms that these components, which contain an aniline‐like structural motif, are responsible for the observed background reaction.

Using cells expressing LmrR_V15pAF or LmrR supplemented with pAF showed a somewhat higher yield, indicating a catalytic effect, albeit quite small. In contrast, when using cells containing the evolved variant LmrR_V15pAF_RMH, the hydrazone products **3 a**,**b** were obtained with significantly higher yield of 62 % and 82 % yield in MMV and 31 % and 73 % in buffer, respectively. These results show that LmrR_V15pAF_RMH catalyses the hydrazone formation in the cell. The lower yields obtained for **3 a** reflect the lower reactivity of **1 a** due to the strongly electron donating ‐OH substituent. Cells expressing the corresponding tyrosine mutant, LmrR_V15Y_RMH, gave rise to a significantly reduced yield of 39 % for **3 b** (Figure S15). This observed drop in the yield observed for the tyrosine variant is consistent with previous results reported with isolated enzyme and further supports that under the expression conditions the catalytically active pAF residue is incorporated efficiently in LmrR_V15pAF_RMH.^[24]^

Next, the biocatalytic synthesis of aromatic aldehydes in *E. coli* K‐12 MG1655 cells was investigated. We focused on production of benzaldehyde and *p‐*hydroxybenzaldehyde, using three different approaches:

- Carboxylic acid reductase (CAR) from *Nocardia iwensis*, along with its one time activator phosphopantetheinyl transferase (SFP) from *Bacillus subtilis*, reduces benzoic acids into their corresponding benzaldehydes in a NADPH and ATP dependent fashion.[^39^, ^40^] A codon‐optimised gene for CAR‐SFP was harboured on a pETDuet‐1 vector to achieve overexpression in *E. coli* K‐12 MG1655.
- Through a combination of feruloyl CoA synthase (encoded by *Atfcs*) and enoyl CoA hydratase/aldolase (encoded by *Atech*) from *Amycolatopsis thermoflava* N1165 *p‐*coumaric acid is converted into *p‐*hydroxybenzaldehyde.^[41]^ The conversion proceeds by the initial formation of the SCoA derivative by FCS, followed by the conjugate addition of water by ECH/hydratase and retro‐aldol condensation by ECH/aldolase to produce the corresponding aldehyde.[^42^, ^43^, ^44^] The required enzymes were expressed heterogeneously using a pETDuet‐1 vector harbouring the *Atfcs_Atech* genes.
- 5‐Hydroxymethylfurfural (HMF) oxidase (HMFO) from *Methylovorus sp. strain MP688*, a flavoprotein oxidase uses molecular oxygen to oxidize various aromatic alcohols,[^45^, ^46^] with concomitant formation of H_2_O_2_. The HMFO gene was harboured on a pBAD vector.

The respective enzymes were expressed at 24 °C for 16 h in MMV medium and the required substrates of 5 mM final concentration were added immediately to FCS‐ECH (**5**) and CAR‐SFP (**4 a**,**b**) containing cells (Scheme 1). For HMFO containing cells, 5 mM substrate **6 a**/**b** was added after 16 h of expression and then reacted for 2 h (Scheme 1).

Both FCS‐ECH/*E. coli RARE*, starting from *p‐*coumaric acid, and CAR‐SFP/*E. coli RARE*, from *p‐*hydroxybenzoic acid, produced a good yield of *p‐*hydroxybenzaldehyde. In contrast, the yield of the reduction of benzoic acid to benzaldehyde catalysed by CAR‐SFP/*E. coli RARE* was significantly lower. FCS‐ECH does not accept cinnamic acid as substrate. The whole cell HMFO catalysed oxidation of benzyl alcohol and *p‐*hydroxybenzyl alcohol gave **3 a** and **3 b** in 30 and 79 % yield, respectively (Figure S3).

Having demonstrated that both LmrR_V15pAF_RMH catalysed hydrazone formation and the production of its substrates benzaldehyde and *p‐*hydroxybenzaldehyde can be achieved in *E. coli* RARE cells, we sought to combine these processes to create the in vivo biocatalytic cascade. Initial attempts to integrate the genes that encode CAR‐SFP or FCS‐ECH, LmrR_V15TAG_RMH and the orthogonal translation system on two plasmids proved unsuccessful due to the large size of the *CAR‐SFP* and *FCS‐ECH* genes. For this reason, we then constructed the system by integrating three plasmids: pETDuet‐1 harbouring either the *CAR‐SFP* or *FCS‐ECH* genes, pET28b+ harbouring the gene for LmrR_V15pAF_RMH and pDULE‐para‐aminoPhe 2 containing the genes for the orthogonal translation system. The three plasmids were co‐transformed into *E. coli* RARE.

**Figure 2:**
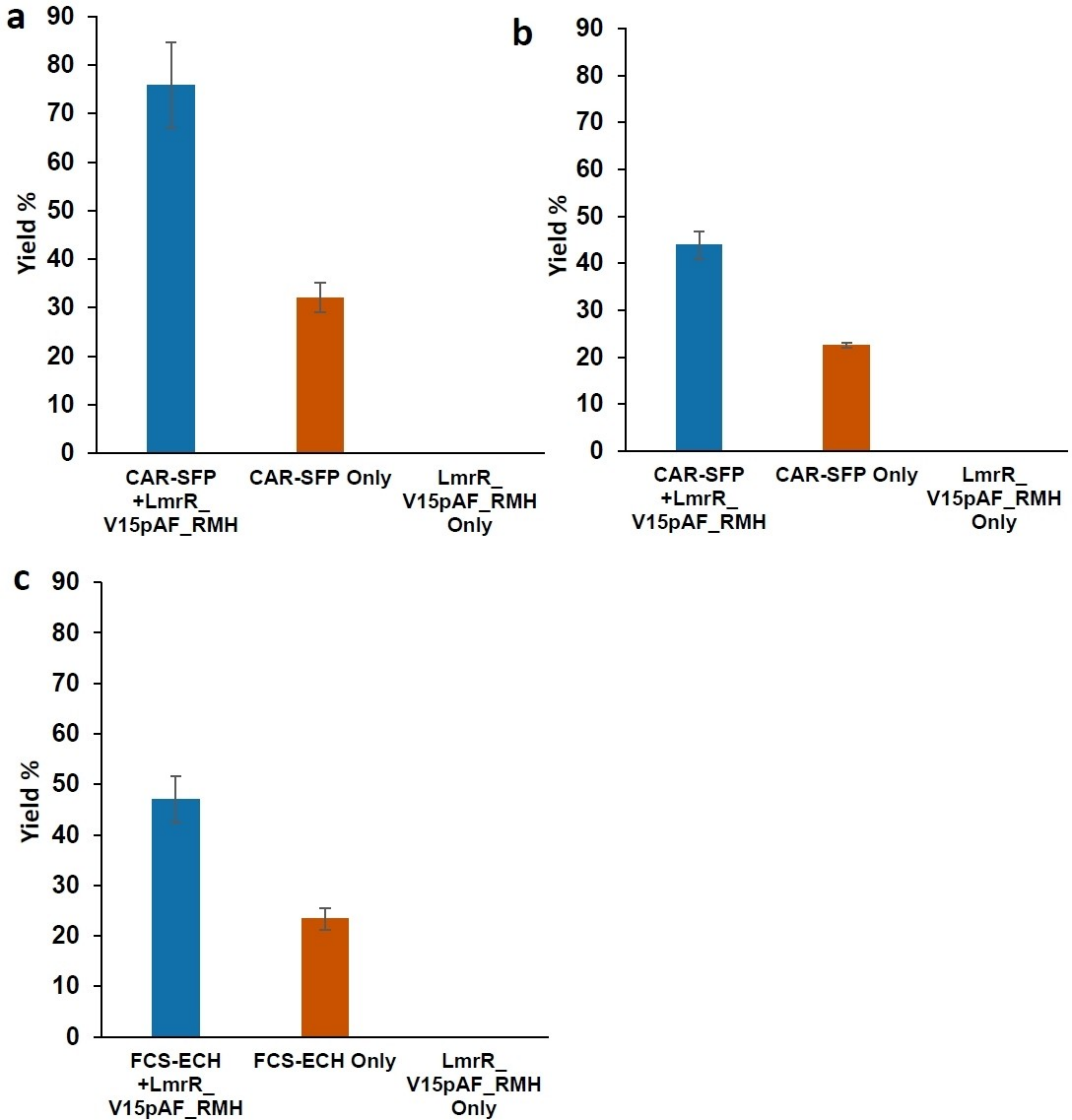
Yield of hydrazone products in in vivo cascade reactions using three plasmid systems. a) Yield of **3 b** with the CAR‐SFP/LmrR_V15pAF_RMH system in *E.coli* K12 MG1655 (RARE). Conditions: 5 mM of **4 b** and 50 μM **2** for 24 h at 24 °C/135 rpm. b) Yield of **3 a**, using **4 a** as substrate. Conditions as under (a). c) Yield of **3 a** with FCS‐ECH/LmrR_V15pAF_RMH system. Conditions: 5 mM **5** and 50 μM **2**, for 6 h at 24 °C/135 rpm. Error margins represent standard deviations from experiments performed in triplicate.

First, the different cell variants were tested in minimal media in the absence of NBD−H, to evaluate the production of aldehyde in the presence of all genes. In all cases, aldehyde was still produced, although the yields sometimes differed as compared to the single plasmid system (Figure S3). The in vivo cascade hydrazone formation reactions with CAR‐SFP and FCS‐ECH were performed using cells from a 2.5 mL cell culture that were resuspended in freshly prepared MMV medium (Figure 2). In buffer, no reaction was observed, presumably since the required co‐factors for CAR or FCS‐ECH are not available at sufficient concentrations. The cascade reaction was started by adding NBD−H (final concentration 50 μM) and aldehyde precursor (final concentration 5 mM). After 24 h at 24 °C, the whole cells expressing only LmrR_V15pAF_RMH showed no reactivity, consistent with an absence of aldehyde product. Only CAR, in the absence of LmrR_V15pAF_RMH, resulted in 28 % yield of the hydrazone, which was not unexpected in view of the background reaction observed in MMV (see above). CAR with LmrR_V15pAF_RMH showed good reactivity with a hydrazone yield of 57 % after 3 h in whole cells with benzoic as aldehyde precursor, and also gave rise to formation of the corresponding hydrazone product with *p*‐hydroxybenzoic as aldehyde precursor. Since significantly higher yields of product were observed when both CAR and LmrR_V15pAF_RMH were present, as compared to CAR alone, it can be concluded that the hydrazone product is formed predominantly from the combination of the heterologous biosynthesis of aldehyde and hydrazone formation catalysed by the artificial enzyme. Using whole cells expressing FCS‐ECH/LmrR_V15pAF_RMH also afforded good yields of hydrazone products where *p‐*coumaric acid was used as aldehyde precursor, which were also significantly higher than the background due to medium.

**Figure 3:**
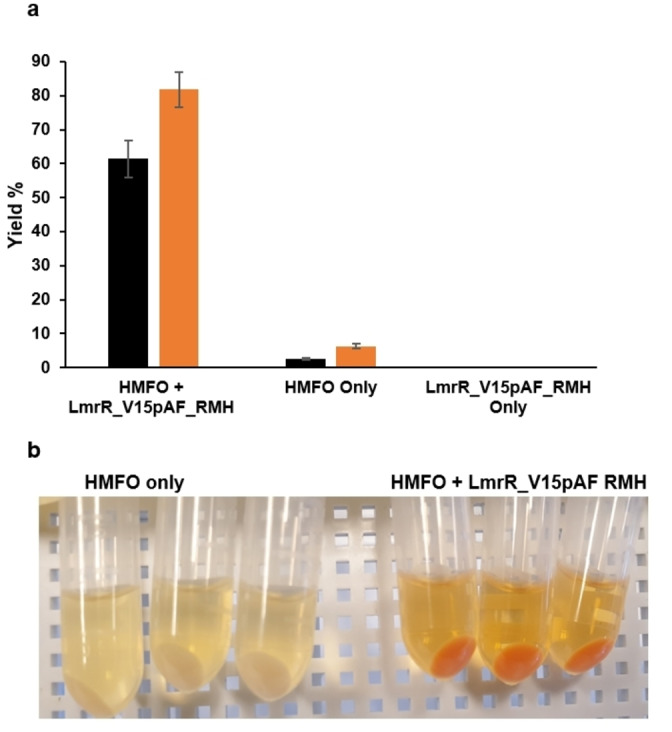
a) The in vivo cascade hydrazone formation reaction with HMFO/LmrR_V15pAF_RMH in 2.5 mL cell culture in phosphate buffer (pH 6.5) using 50 μM **2** and 5 mM **6 b** at 24 °C with 135 rpm for 1 h (black) to 2 h (orange). Error margins represent standard deviations from experiments performed in triplicate. b) A photograph of hydrazone formation after 2 h with HMFO_LmrR_V15pAF_RMH (right) compared to HMFO only (left).

For the in vivo cascade of HMFO and LmrR_V15pAF_RMH in cells, a two‐plasmid system comprising the pBAD vector, which contains the genes for HMFO and LmrR_V15pAF_RMH, and pDULE‐para‐aminoPhe 2 was used, permitted by the smaller size of the *HMFO* gene. The cascade alcohol oxidation and hydrazone formation were first tested in vitro, using purified HMFO and LmrR_V15pAF_RMH resulting in excellent yield of the product. It was determined that the H_2_O_2_ produced by HMFO does not negatively affect the reaction, since the addition of catalase did not significantly alter the outcome (Figure S12). Then, we performed the in vivo oxidation and hydrazone formation cascade reactions using the whole cells expressing HMFO and LmrR_V15pAF_RMH. The reactions were set up with NBD−H (final concentration 50 μM) and benzyl alcohol (**6 b**, final concentration 5 mM) in cells for 2 h in phosphate buffer pH 6.5. The cells expressing HMFO and LmrR_V15pAF_RMH gave the corresponding hydrazone product **3 b** with 81 % yield after 2 h. In contrast, the control experiment with cells only expressing HMFO gave only 6 % yield, which is the background reaction. Using *p‐*hydroxybenzyl alcohol (**6 a**) afforded 41 % yield of the hydrazone product after 20 h. Those results suggest the successful and efficient in vivo cascade pathway by the combination of a natural enzyme and an artificial enzyme. Since the HFMO/LmrR_V15pAF_RMH cascade gave such a large difference compared to background reaction, it is possible to observe the difference in reactivity by visual inspection (Figure 3b).

To confirm that the catalysis occurs in the cell and that the cells remain intact during and after catalysis a recycling experiment was performed. In the first round, we performed the cascade reaction by using 5 mM **6 b** and 50 μM **2**. The reaction was kept at 24 °C/135 rpm for 3 h, resulting in 63 % yield of the hydrazone product **3 b** (Figure S21). The cells were then washed with 50 mM Kpi buffer at pH 6.5 to remove all extracellular components, resuspended in reaction buffer and 5 mM **6 a** and 50 μM **2** were added. After reaction for 16 h, the corresponding product **3 a** was obtained in 22 % yield, accompanied by approximately 10 % residual product from the first round. This experiment confirms that the cells remain intact and that both the HMFO and the artificial enzyme remained inside the cells during catalysis. The in vivo cascade reaction was performed at a preparative scale (26 mg **2**) and the product **3 b** was isolated in 24 % yield after column chromatography.

The viability of the remaining cells after catalysis was tested by centrifuging and resuspending in 1 ml LB, after which serial dilutions were plated on agar containing the required antibiotics. 336 colonies of *HMFO/LmrR_V15pAF_RMH*/*E. coli* RARE were still present on plates with cells diluted to 10^−6^ (Figure S22). This means that the reaction conditions, that is, in the presence of both the canonical and artificial enzymes, as well as the substrates and the hydrazone product, are well tolerated.

## Conclusion

In conclusion, we have created in vivo biocatalytic cascades in *E. coli* that comprise a combination of natural and artificial enzymes. These results show that a heterologous biosynthetic pathway can be augmented with a new‐to‐nature catalytic reaction for the production of novel compounds. While this work represents a proof of principle, it is a step towards the creation of a hybrid metabolism, which combines multiple natural and artificial enzymes in living cells to produce complex molecules from simple bio‐based starting materials.

## Conflict of interest

The authors declare no conflict of interest.

1

## Supporting information

As a service to our authors and readers, this journal provides supporting information supplied by the authors. Such materials are peer reviewed and may be re‐organized for online delivery, but are not copy‐edited or typeset. Technical support issues arising from supporting information (other than missing files) should be addressed to the authors.

Supporting Information

## Data Availability

The data that support the findings of this study are available in the supplementary material of this article.
