# Supplementary material for: In Vivo Biocatalytic Cascades Featuring an Artificial‐Enzyme‐Catalysed New‐to‐Nature Reaction
Source: Angew Chem Int Ed Engl. 2022 Nov 30;62(1):e202214191. doi: 10.1002/anie.202214191 (PMC10100225; doi:10.1002/anie.202214191)
Supplement: Supplementary file 1 — Supporting Information [file ANIE-62-0-s001.pdf]

Angewandte Chemie
Eine Zeitschrift der Gesellschaft Deutscher Chemiker

Supporting Information

In Vivo Biocatalytic Cascades Featuring an Artificial-Enzyme-Catalysed New-to-Nature Reaction

*L. Ofori Atta, Z. Zhou, G. Roelfes**

Supporting information

I

Table of Contents

Supplementary tables 2
Supplementary figure 2
Methods and Materials 3
Protein biosynthesis 4
HPLC Chromatogram for aldehydes 7
Protein Purification 7
Trypsin digest 8
Mass Spectrometry Analysis 9
Uv-vis spectroscopy 13
UPLC-MS Analysis 13
Hydrazone formation reaction *in vivo* / *in vitro* 14
Background reaction of hydrazone formation 17
HPLC Chromatogram for hydrazone 18
Reductive amination using $NaCNBH_3$ 20
Recycling Experiment 24
Cell viability test after catalysis 25
Plasmid maps of plasmids used in this study 30
Calibration Curves for Aldehydes 31
Chemical synthesis and calibration curves of hydrazone products 3a and 3b 31
NMR spectra 33
Scale up of in vivo hydrazone reaction using LmrR_V15pAF_RMH/HMFO system 34
References 36

Supplementary tables

E. coli Strains	Plasmids
K-12 MG1655 (RARE) for expression	pETDuet-1 harbouring *CAR-SFP*
NEB 5 alpha for cloning	pETDuet-1 harbouring *FCS-ECH*
NEB 10 beta for cloning	pET28b+ harbouring LmrR-V15pAF_RMH
	pBAD harbouring HMFO and LmrR-V15pAF_RMH
	pDULE 2 harbouring pAF OTS

Table S1. Bacteria strains and plasmid used in this study.

Protein Sample	Expression plasmid/OTS	k_{cat}/K_m (app) M^{-1}/s^{-1}
LmrR_V15pAF_RMH	pEVOL_pAzF	70
LmrR_V15pAF_RMH	pDULE 2 pAF	54

Table S2. Comparison of catalytic efficiency in the hydrazone formation reaction of **1a** with **2** to form **3**, by artificial enzymes produced using direct incorporation of pAF and indirect incorporation of pAF into LmrR_V15X RMH using the hydrazone formation reaction.[1] Based on the relative activitiy it is concluded that the pAF incorporation efficiency is ~80% when using the direct incorporation method. k_{cat}/K_m **(app)** M^{-1}/S^{-1} = catalytic efficiency **Extinction Coefficient (ε)** =29,585 M^{-1} cm^{-1} .

Supplementary figure

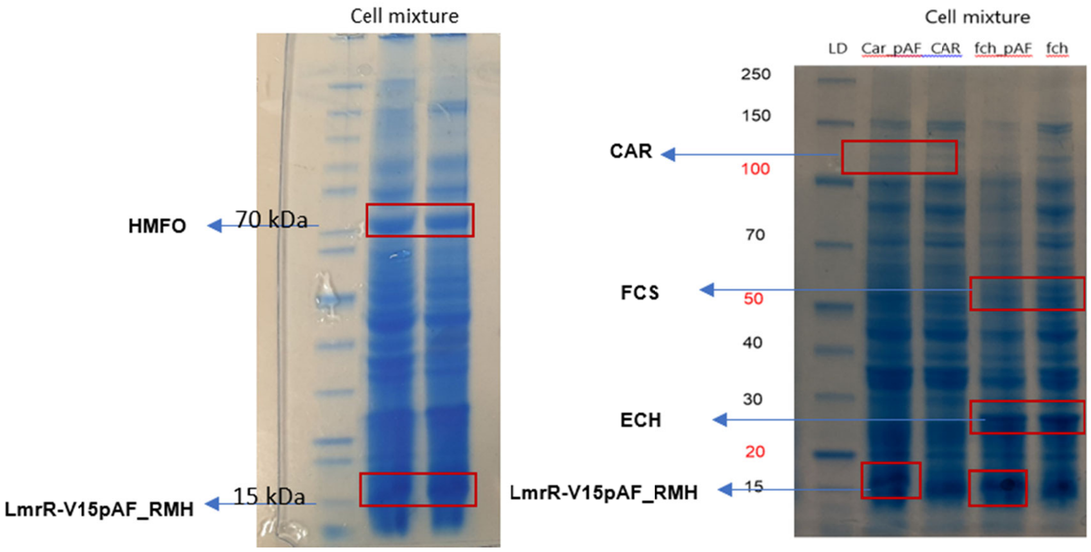

Figure S1. Expression of LmrR_V15pAF_RMH, CAR, FCS-ECH and HMFO after integration of the plasmids in E. coli RARE. CAR showed a low expression, most likely caused by its large gene size of 4000 bp, making it difficult for RNA polymerase to synthesise the protein.

Methods and Materials

Strains and Plasmids: *E. coli* strains and plasmids used in this study are listed in Table S1. Molecular biology techniques were performed using standard practices unless otherwise stated. Molecular cloning and vector propagation were performed in K-12 MG1655**.** The *CAR* gene from *Nocardia iowensis* and *SFP* gene from *Bacillus subtilis*, were synthesized and codon-optimized for expression in E. coli (GenScript USA). The pDULE plasmid harbouring pAF synthase\tRNA genes was purchased from Addgene (pDule2-para-aminoPhe was a gift from Ryan Mehl, Addgene plasmid #85503)[2], the pBad plasmid harbouring *HMFO* was a gift from the Fraaije group (University of Groningen). Purified HMFO used in this study was a gift from Gecco Biotech (www.gecco-biotech.com).

Cell culture conditions: A standard recipe for Lysogenic Broth (LB) medium was used (10 g/L Bacto tryptone, 5 g/L Yeast extract, 10 g/L NaCl and 15 g/L Agar for LB-Agar plates). MMV medium was prepared with the following recipe, 5.3 g/L $Na_2HPO_4 \times 12\ H_2O$, 1.4 g/L KH_2PO_4, 0.2 g/L $MgSO_4 \times 7\ H_2O$, 1.0 g/L $(NH_4)_2SO_4$. The following components were sterile-filtered using 0.2 µm filter before adding the medium: 1 ml/L vitamin solution (1000 ×), 5 ml/L trace solution (200 ×), 4 ml/L glucose (50%). M9 medium was prepared using the following recipe, 56 g/L of M9 salt (mix by Sigma) and a 0.2 µm filter was used to filter the following component before addition 100 µl of 1 M $CaCl_2$, 2 mL of 1 M $MgSO_4$, 8 mL of 50% glucose solution, 5 mL of 4% vitamin B1 (thiamine), 50 mL of 20% Cas-amino acids.

Minimal media with vitamins

The minimal media with vitamins used in this work was prepared as follows. The content per litre had 5.3 g $Na_2HPO_4 \cdot 12H_2O$, 1.4 g KH_2PO_4, 0.2 g $MgSO_4 \cdot 7H_2O$, 1.0 g $(NH_4)_2SO_4$, 1 ml vitamin solution and 5 ml of a trace element solution, 4 ml glucose (50%) and Antibiotics . The trace element solution per litre contained 780 mg $Ca(NO_3)_2 \cdot 4H_2O$, 200 mg $FeSO_4 \cdot 7H_2O$, 10 mg $ZnSO_4 \cdot 7H_2O$, 10 mg H_3BO_3, 10 mg of $CoCl_2 \cdot 6H_2O$, 10 mg $CuSO_4 \cdot 5H_2O$, 4 mg $MnSO_4 \cdot H_2O$, 3 mg $Na_2MoO_4 \cdot 2H_2O$, 2 mg $NiCl_2 \cdot 6H_2O$, and 2 mg $Na_2WO_4 \cdot 2H_2O$ [3]. The vitamin solution consisted of 2.2 mg Biotin, 2.2 mg folic acid, 220 mg p-aminobenzoic acid, 220 mg Riboflavin, 440 mg pantothenic acid, 440 mg niacinamide, 440 mg pyridoxine×HCl, 440 mg, thiamine×HCl per litre [4].

Protein biosynthesis

For experiments testing the direct incorporation of pAF into LmrRV15TAG, pDULE vector harboring pAF synthase\tRNA and pET17b+ vector harboring LmrRV15TAG were co-transformed into *E. coli* k-12 MG1655 (RARE). Culture media were supplemented with 100 µg/L ampicillin and 10 µg/L streptomycin to provide selective pressure for plasmid maintenance. Except for cultures grown in LB medium, overnight pre-cultures were pelleted and re-suspended in either M9 medium or MMV medium. This step was done to ensure that nutrient rich LB medium was removed. Experimental cultures were then initiated by adding 2 % (v/v) of re-suspended pre-cultures into 50 ml either M9 or MMV medium while 1 % (v/v) pre-culture was added to LB medium. Gene expression was then induced with 1 M isopropyl-β-d-1-thiogalactopyranoside (IPTG) (1 mM final concentration) and 20 % L- arabinose (0.02 % final concentration) between an OD_{600} of 0.4 and 1. During induction, 1 M pAF (1 mM final concentration) was added in solid form, incubated at 24 °C, and agitated at 135 rpm for 48 hrs. These experiments were performed in 250 ml baffled Erlenmeyer flasks for better aeration.

**Media/Sample**	**Strain** *E.Coli* **K-12 MG1655**	**pAF**	**Temperature**	**Time**
**LB**	**RARE**	**1mM**	**30 °C**	**16 h**
**LB control**	**RARE**	**No pAF added**	**30 °C**	**16 h**
**M9**	**RARE**	**1 mM**	**30 °C**	**16 h**
**M9 control**	**RARE**	**No pAF added**	**30 °C**	**16 h**
**MMV**	**RARE**	**1 mM**	**24 °C**	**48 hours**
**MMV control**	**RARE**	**No pAF added**	**24 °C**	**48 hours**

Table S3. Expression conditions for pAF incorporation into LmrR_V15X_RMH.

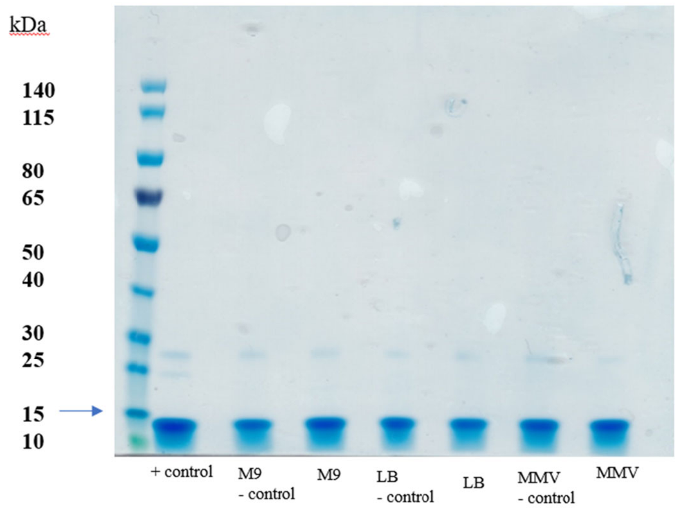

Figure S2. SDS-PAGE analysis of pAF incorporation into LmrR-V15X_RMH. Controls without pAF added do yield protein, which is caused by misincorporation of canonical amino acids due to the promiscuity of the orthogonal translation system.

Metabolites analysis:

For aromatic aldehydes synthesis, experiments were performed in 250/500 ml Erlenmeyer flasks (Sigma), using 50 mL MMV, supplemented with 100 µg ampicillin. Experimental cultures were initiated by adding 1 % (v/v) inoculum volumes of overnight pre-culture to 50 ml volume of MMV, incubated at 37 °C, and agitated at 135 rpm.

Expression was induced with 1 mM isopropyl-β-d-1-thiogalactopyranoside (IPTG) or 0.02 % arabinose between optical density 600 (OD_{600}) of 0.8−1.0. The cultures were incubated at 24 °C and 135 rpm for 16 h. Cultures were spun down at 4000 rpm for 10 mins, pellets were resuspended with fresh MMV and 5 mM of substrate was added at 24 °C and 135 rpm for 16 h. For benzaldehyde producing cultures, samples were spun down and filtered with a 0.2 µM filter before HPLC analysis. In cultures producing para hydroxy benzaldehyde, the products were extracted with ethyl acetate.

HPLC analysis using an Agilent series instrument equipped with a diode array detector was used to detect products at wavelengths of 247 nm and 281 nm for benzaldehyde and para-hydroxy benzaldehyde, respectively. A kinetex-C18 column was used and the gradient method used the following solvents:

(A1) acetonitrile + 0.1% trifluoroacetic acid (TFA); (B1) water +0.1% TFA. The gradient started with 5% Solvent A1 and 95% Solvent B1 with a flow rate of 1.0

mL/min, and all compounds of interest eluted within 30 mins. The column temperature was maintained at 25°C.

Gene	Strain *E.Coli* K-12 MG1655	Substrate (5 mM)	Temperature	Time (hours)
CAR_SFP	RARE	Benzoic Acid	24°C	16 h / 48 h
CAR_SFP	RARE	4 hydroxy-benzoic Acid	24 °C	16 h / 48 h
FCS-ECH	RARE	4-coumaric acid	24 °C	16 h / 48 h
HMFO	RARE	benzyl alcohol	24 °C	16 h / 48 h
HMFO	RARE	4 hydroxy-benzyl alcohol	24 °C	16 h / 48 h

Table S4. Expression conditions for *in vivo* aldehydes production using *E.coli* K12 MG1655 (RARE).

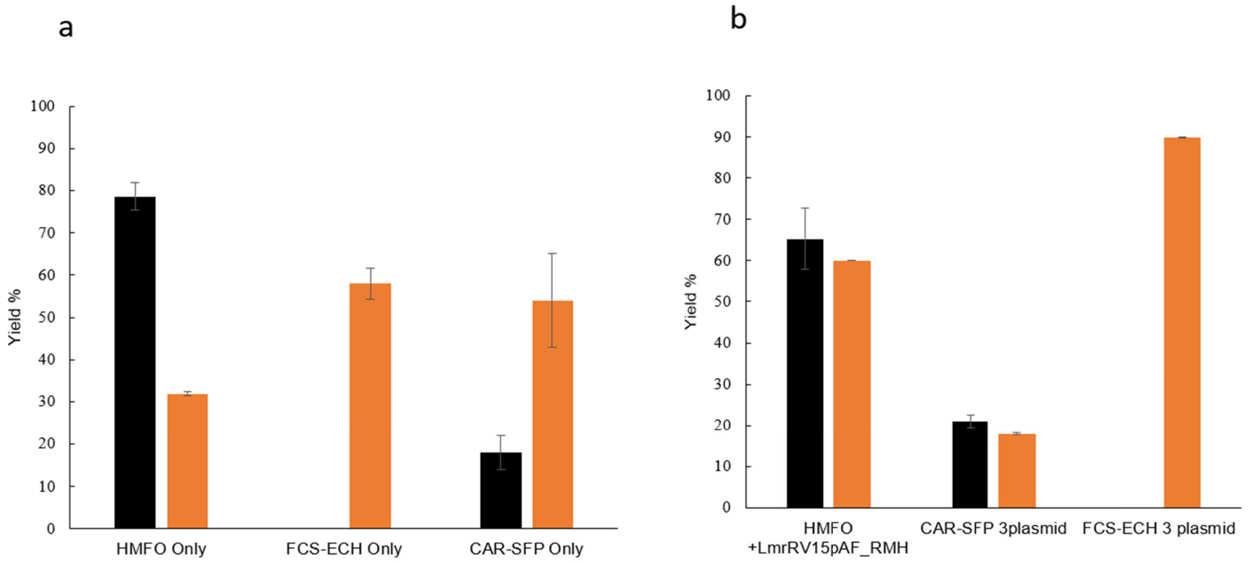

Figure S3. *In vivo* production of aldehydes before and after the integration of natural enzymes with LmrR-V15pAF_RMH in *E.coli* K12 MG1655 (RARE). **(a)** Benzaldehyde (black) was produced by HMFO and CAR-SFP and para hydroxy benzaldehyde (orange) was produced by HMFO, CAR-SFP and FCS- ECH. Expression was done at 24℃/135 rpm for 24 h. (**b**) Aldehydes production using 3 plasmid system (CAR-SFP, FCS=ECH) and 2 plasmid system (HMFO), after the integration of natural enzymes with artificial enzyme (LmrR-V15pAF_RMH). Expression was done at 24℃/135 rpm for 48 h.

HPLC Chromatogram for aldehydes

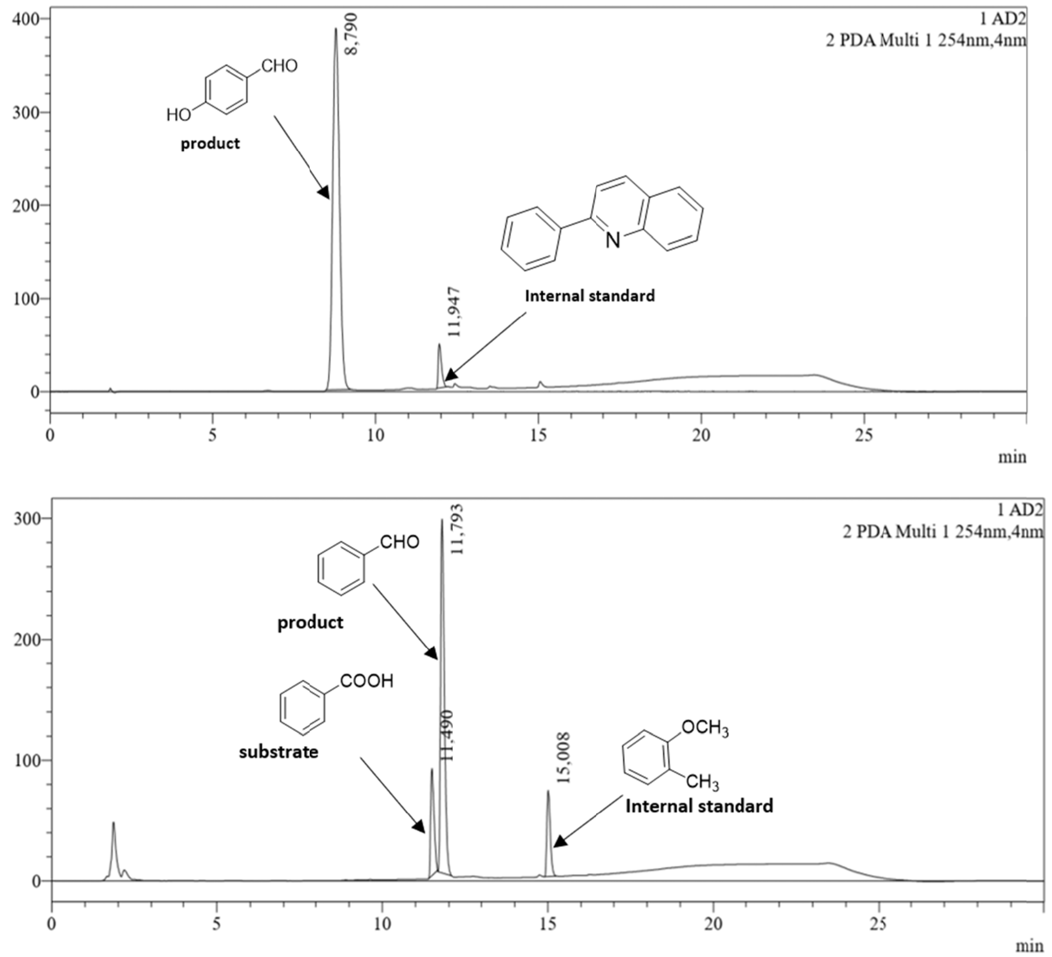

Figure S4. HPLC chromatogram of aldehydes biosynthesised in *E.coli* K12 MG1655 (RARE). (**a**) p-OH benzaldehyde product **3a** peak detected at 8,7 min while the internal standard 2-Phenylquinoline had a retention time around 11,9 min. (**b**) Benzaldehyde product **3b** peak detected at 11,7 min while the benzoic acid substrate peak had a retention time of 11,4 mins. Here 2-methylanisole was used as an internal standard.

Protein Purification:

Sodium phosphate buffer (in milliQ water) was prepared with the following recipe: 50 mM of Na_2HPO_4; 12 H_2O, 150 mM of NaCl at pH 8.The buffer was filtered with 0.2 µm sterile filters. After 48 hours of expression at 24 °C, cells were harvested by centrifugation (6,000 rpm., JA10, Beckman) for 20 min, at 4 °C. The cell pellet was resuspended in 20 ml Sodium phosphate buffer and half a tablet “mini complete protease inhibitor cocktail” (Roche) was added. The solutions were sonicated on ice (8 min, pulse of 10 s ON and 15 s OFF, amplitude of 70 %).

The lysed cells were incubated on ice with 0.1 mg /ml of DNase I and 10 mM of $MgCl_2$ for 30 min. The mixture was spun down with rotor JA17(Beckman), 12,000 rpm, for 60 min, 4 °C. The supernatant was filtered using a 0.45 µm filter and was

loaded on a Strep-Tactin column (Strep-Tactin Superflow high capacity), incubated for 60 min at 4 °C, and the protein was purified according to the manufacturer's guidelines. Eluents were collected and dialysed against the reaction buffer (50 mM NaH_2PO_4, 150 mM NaCl, pH 7.4, two times against 1 L). The concentration of the protein was determined by using the calculated extinction coefficient for LmrR, corrected for the absorbance of pAF (ε280 = 1,333 M^{-1} cm^{-1}).

Trypsin digest

Protein for trypsin digest was initially purified by Streptag affinity chromatography and desalted with dialysis. 4 µl of the LmrRv15pAF_RMH protein solution was diluted to 20 µl using a 100 mM ammonium bicarbonate (ABC) solution. 20 µl of a 10 ng/µl trypsin solution in 100 mM ABC was added and incubated overnight at 37 °C. The digestion mixture was acidified by adding 4 µl formic acid and desalted using a C18 stage tip. The eluted peptides were dried in a speedvac and dissolved in 20 µl 0.1% formic acid and injected into the nanoLC system for LCMSMS analysis. Resulting data were analysed using PeaksX software (BioSolutions Inc., Waterloo) Ontario, Canada).

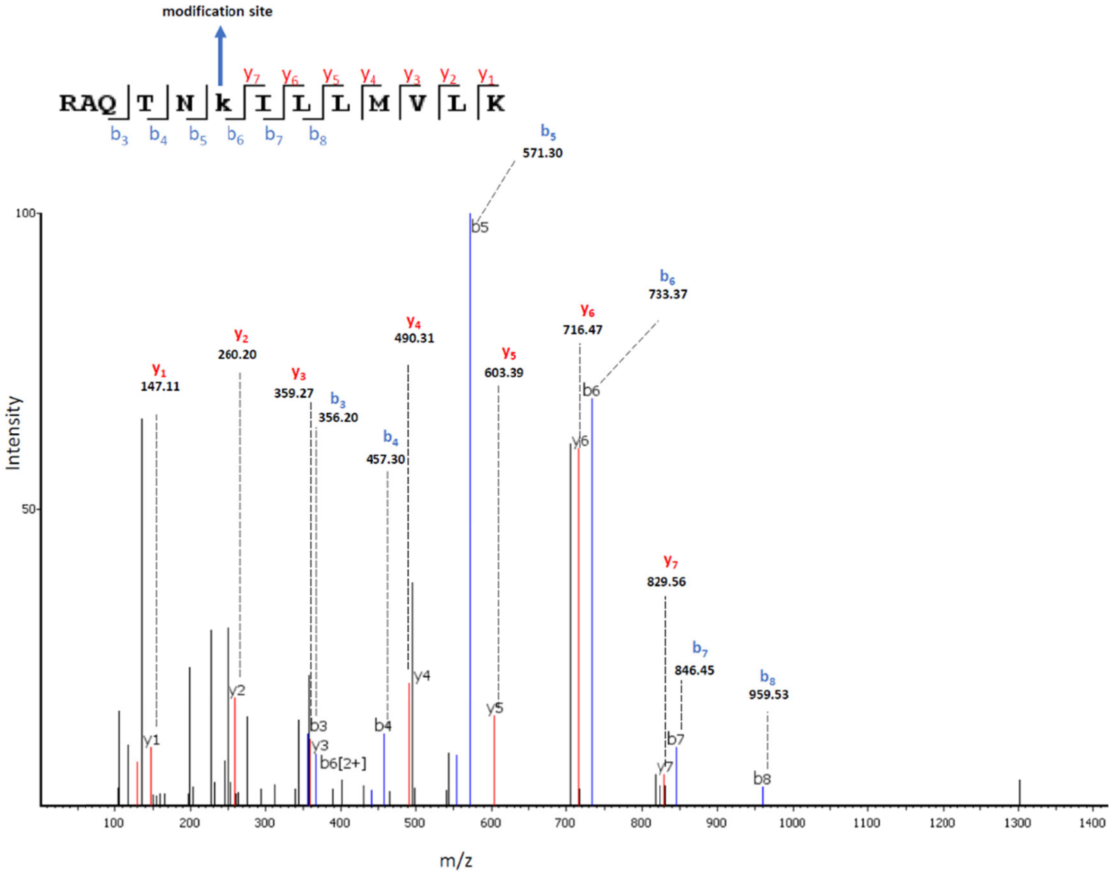

Figure S5. MS/MS results for the trypsin digest of LmrR-V15pAF_RMH expressed in MMV.

Mass Spectrometry Analysis

The mass spectrometry results showed protein expressed in M9 media had phenylalanine misincorporated and protein expressed in LB had a mixture of pAF and phenylalanine incorporated. Protein produced using the MMV media had pAF incorporated with high selectivity. **Expected Mass = 15126 ; Observed Mass = 15126.64000**

Mass spectrometry result sample from MMV media of pAF incorporation into LmrR_V15X_RMH

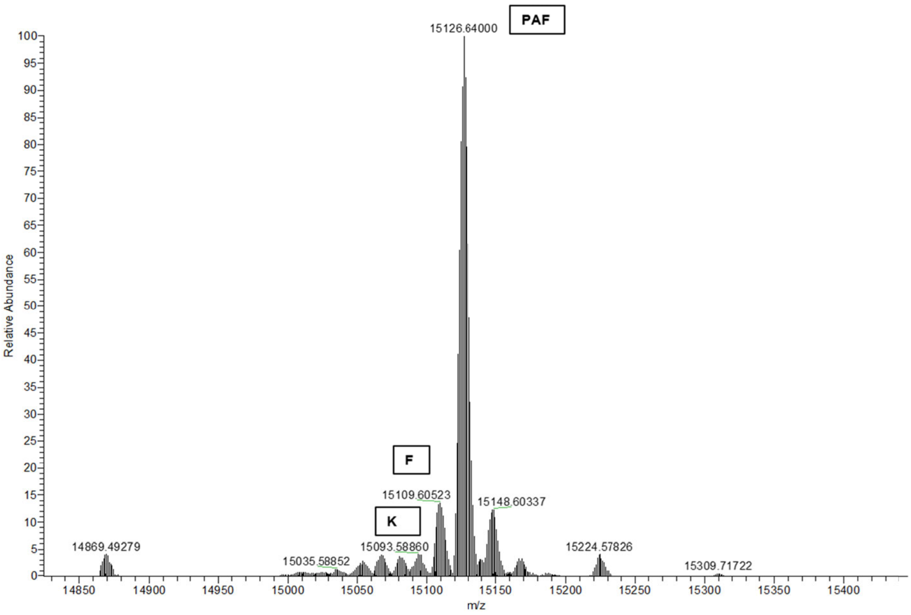

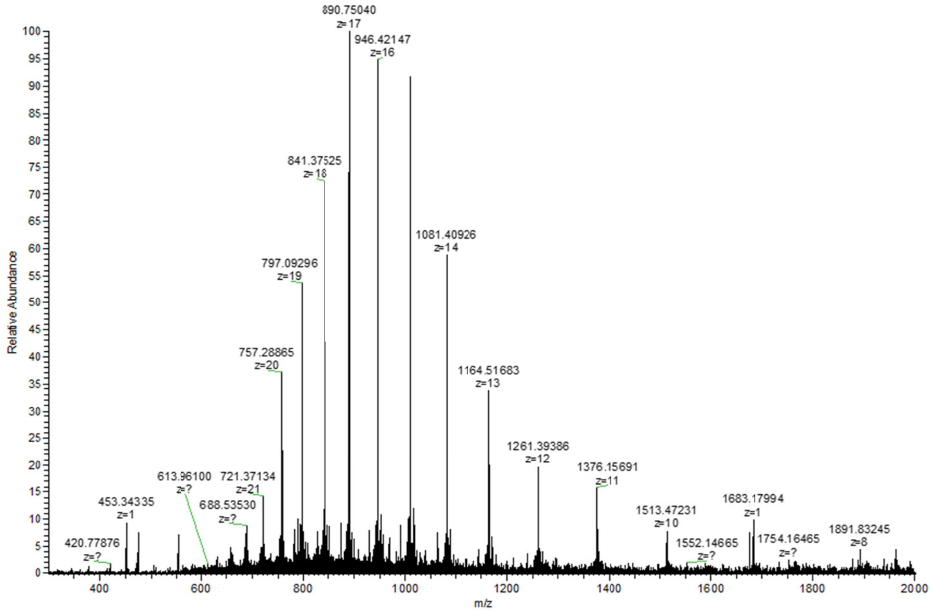

Figure S6. Mass of LmrR-V15pAF_RMH expressed in MMV. After deconvolution, an observed mass of 15126.64, which corresponds to the expected mass of LmrR-V15pAF_RMH, was detected. Although expression in this media gives some misincorporation of phenylalanine (F) and Lysine (K), around 80 % of incorporated amino acid was pAF (unnatural amino acid).

Mass spectrometry result sample from M9 minimal media of pAF incorporation into LmrR_V15X_RMH

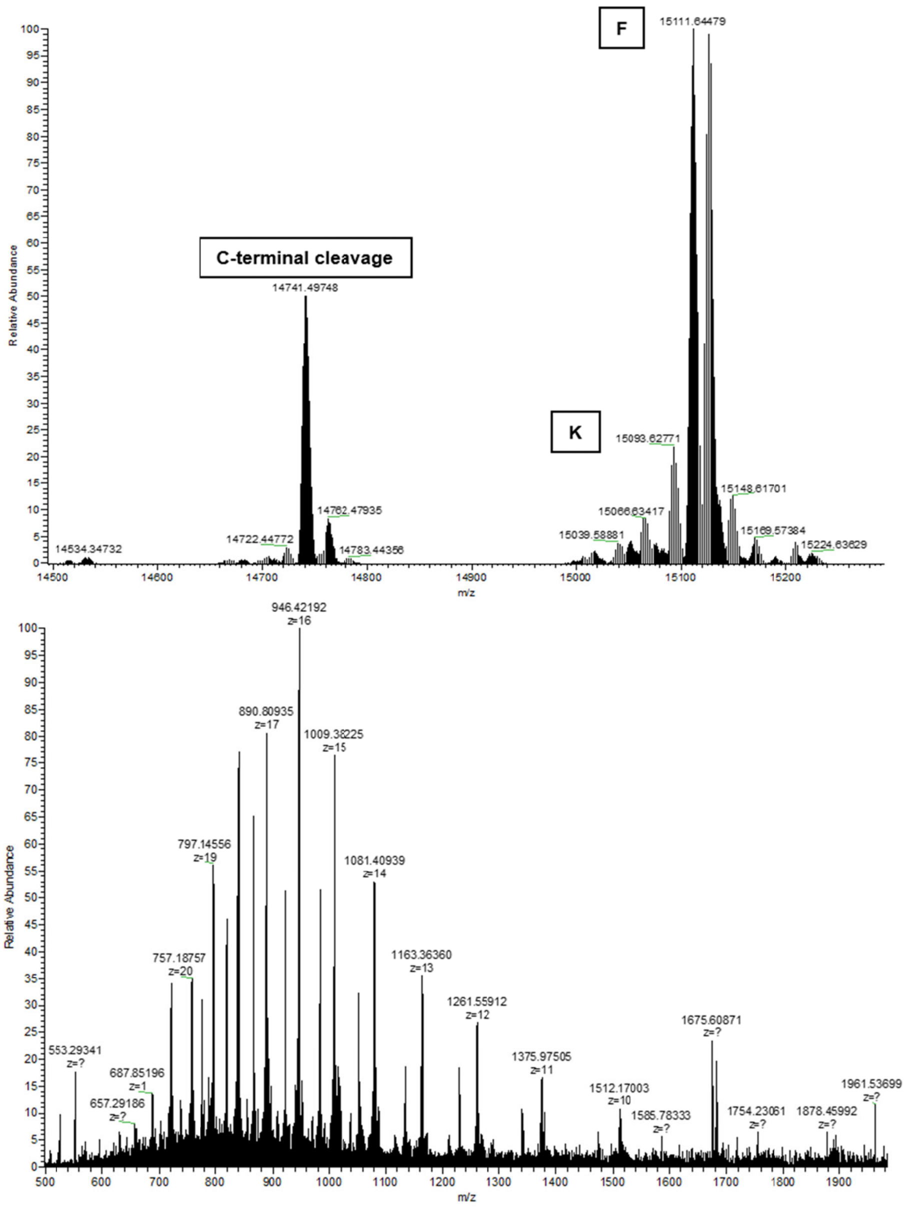

Figure S7. Mass of LmrR-V15X_RMH expressed in M9 minimal media. After deconvolution, an observed mass of 15111.64 which corresponds to the expected mass of LmrR-V15X_RMH when phenylalanine (F) is incorporation instead of pAF. A mass corresponding to the cleavage of 4 amino acids from the C-terminal was also detected as well as few misincorporation of Lysine (K).

Mass spectrometry result sample from LB media of pAF incorporation into LmrR_V15X_RMH

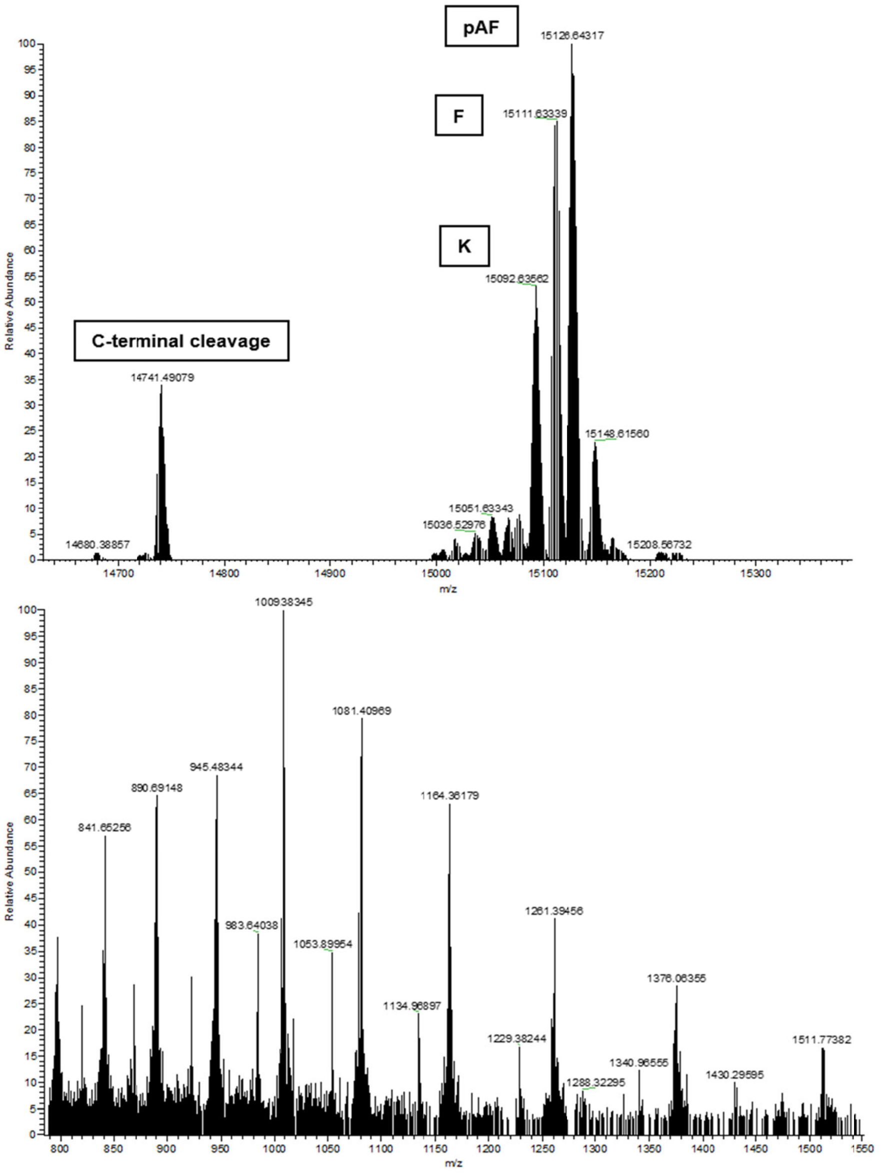

Figure S8. Mass of LmrR-V15X_RMH expressed in LB media. Although after deconvolution an observed mass of 15126.64 which corresponds to the expected mass of LmrR-V15pAF_RMH was detected, there was also a lot of misincorporation of phenylalanine (F) and Lysine (K). The presence of C-terminal cleavage was also detected as it was with M9 sample.

Mass spectrometry result of negative control sample of pAF incorporation into LmrR_V15X_RMH

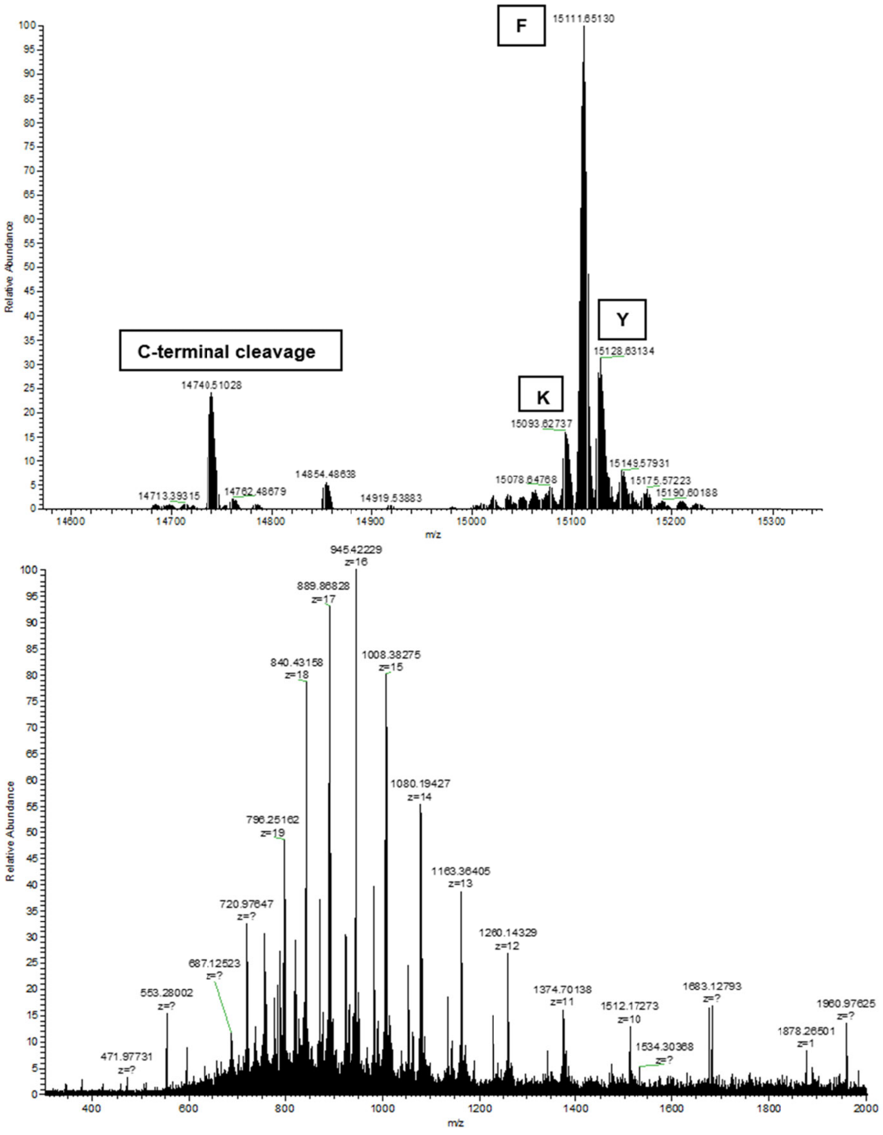

Figure S9. Mass of LmrR_V15X_RMH expressed in MMV without the addition of pAF unnatural amino acid. After deconvolution, an observed mass of 15111.65, which corresponds to the expected mass of LmrR_V15F_RMH was observed which indicates a major misincorporation of phenylalanine (F). Few misincorporation of lysine(K) and tyrosine (Y) were also detected.

All samples analysed had major misincorporation of phenylalanine (F). In LB this misincorporation was due to the fact, it is a rich medium and due the promiscuity of the pAF orthogonal translation system, (OTS) F is incorporated. The situation is the same for M9 minimal media, because it contains cas amino acids. Moreover, in the control sample in MMV where no pAF was added, there was a major misincorporation of F and few of K and Y although MMV is not a nutrient rich media. However, when pAF was added to the MMV culture, pAF became the main

amino acid (unnatural) incorporated. This means that in the presence of pAF in MMV, pAF is more preferred, and for this reason we choose to work with MMV.

Uv-vis spectroscopy

After hydrazone formation reaction with para hydroxy benzaldehyde (**1a**) and NBDH (**2**), the absorbance of 1 ml of product **3a** solution was measured.

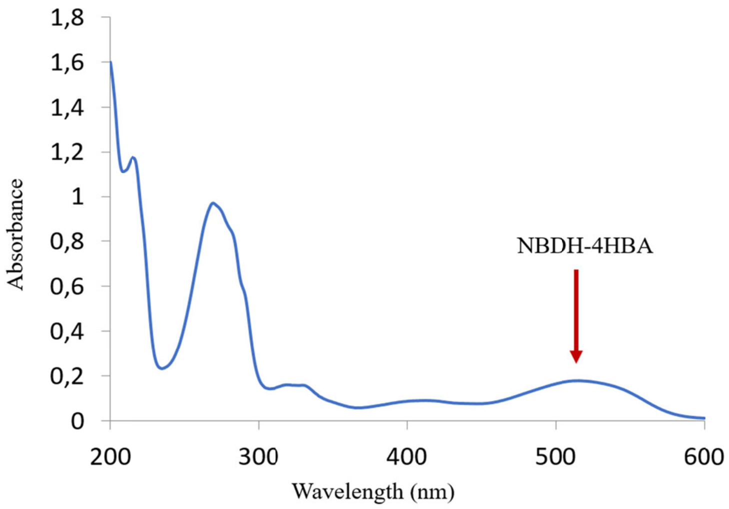

Figure S10. Uv-Vis spectra analysis of product **3a**. The characteristic absorption band of the NBDH-4HBA product (**3a**) was detected at a wavelength of 520 nm.

UPLC-MS Analysis

Reactions samples were pelleted by centrifugation, and aqueous supernatant was collected and filtered with 2 µm filter for UPLC-MS analysis using ACQUITY UPLC® HSS T3 1.8µm. The solvents used were (A) acetonitrile + 0.1% formic acid (TFA); (B) water +0.1% formic acid Run for 20 mins.

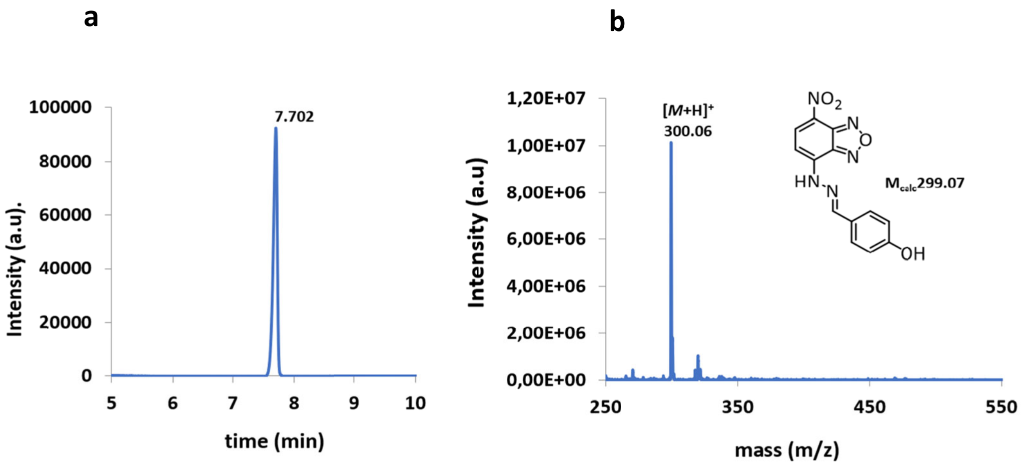

Figure S11. UPLC-MS analysis to confirm the mass of hydrazone product. **(a)** single peak at time 7.7 min corresponded to the retention time of hydrazone on the UPLC. (**b**)The mass of the calculated hydrazone was confirmed.

Hydrazone formation reaction *in vivo* / *in vitro*

Hydrazone formation reaction in vivo: Experimental culture cells were grown in 50 ml MMV supplemented with antibiotics at 37°C, 135 rpm until OD_{600} of 0.4 – 0.7. Gene expression was induced with 1 mM isopropyl-β-d-1-thiogalactopyranoside (IPTG) and 0.02 % L- arabinose. During induction 1 mM pAF was added in solid form, incubated at 24 °C, and agitated at 135 rpm for 48 hrs. Cultures were pelleted and resuspended in newly prepared MMV, to wash away non-incorporated UAA, or in reaction buffer. In the 2.5 mL cell culture with newly prepared MMV medium was added 25 μL NBDH (**2**) (5 mM in DMF) solution (final concentration 50 μM) and 62.5 μL carbonic acid or aldehyde (200 mM in DMF) solution (final concentration 5 mM). Then the mixture was put into the 24 °C shaker at 135 rpm for 1 to 24 hours. The formation of the product was determined by HPLC. Take 500 μL reaction culture into 2 ml Eppendorf, added 50 μL IS (1 mM Fluorescein in DMF), then extracted with 1ml ethyl acetate, after the evaporation, the residue was resuspended in 1200 μL CH_3CN. 200 μL of this solution was placed into HPLC vial and the sample was analyzed by HPLC.

Hydrazone formation reaction in vitro was set-up as follows: To 280 μL of 5 μM HMFO and 4 μM LmrR_V15pAF_RMH in freshly prepared phosphate buffer pH 6.5 was added 10 μL NBDH (1.5 mM in DMF) solution (final concentration 50 μM) and 10 μL carbonic acid (150 mM in DMF) solution (final concentration 5 mM). Then the mixture was put into the 24 °C shaker at 135 rpm for 1 to 3 hours. The yield of the products was determined by HPLC. To the reaction mixture was added 50 μL internal standard (1 mM Fluorescein in DMF), then it was extracted with 200 μL ethyl acetate. After the evaporation, the residue was resuspended in 240 μL CH_3CN. 200 μL of this solution was placed into HPLC vial and the sample was analyzed by HPLC.

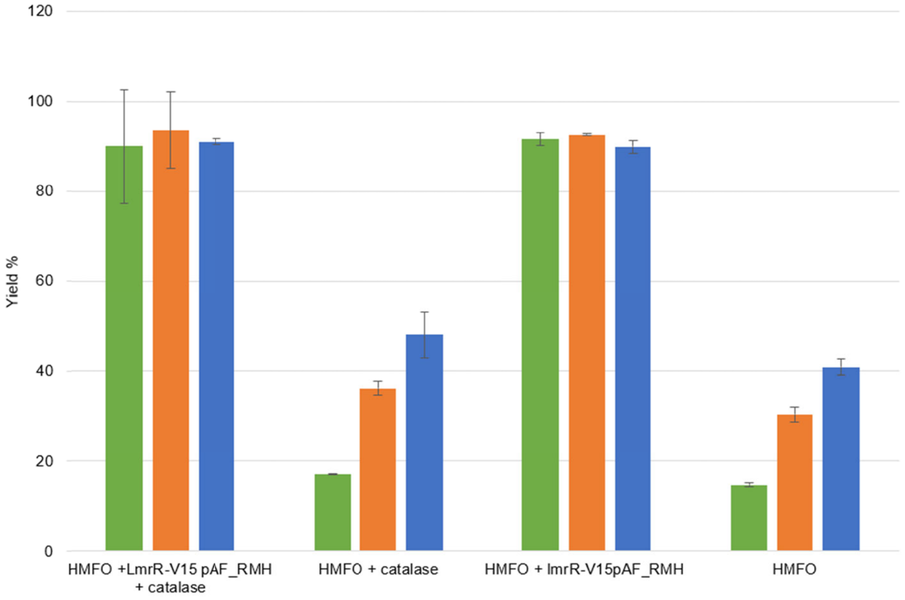

Figure S12. Hydrazone formation by the combination of HMFO and LmrR_V15pAF_RMH *in vitro*.
The in vitro reaction using the combination of HMFO and LmrR-V15pAF_RMH gave 85% yield after 1 h (green). Monitoring at 2 h (orange) and 3 h (blue) didn't give rise to further increased product formation. The control experiments with catalase showed that H_2O_2 has no effect on the cascade reactions.

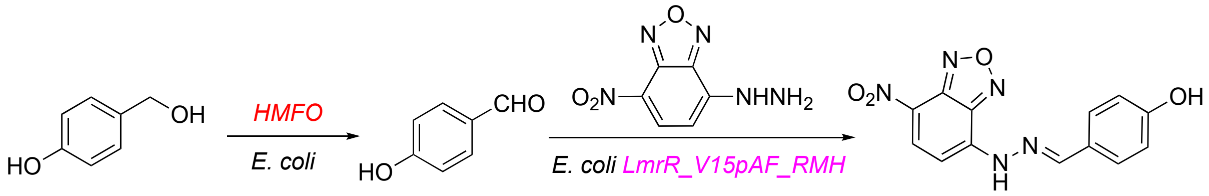

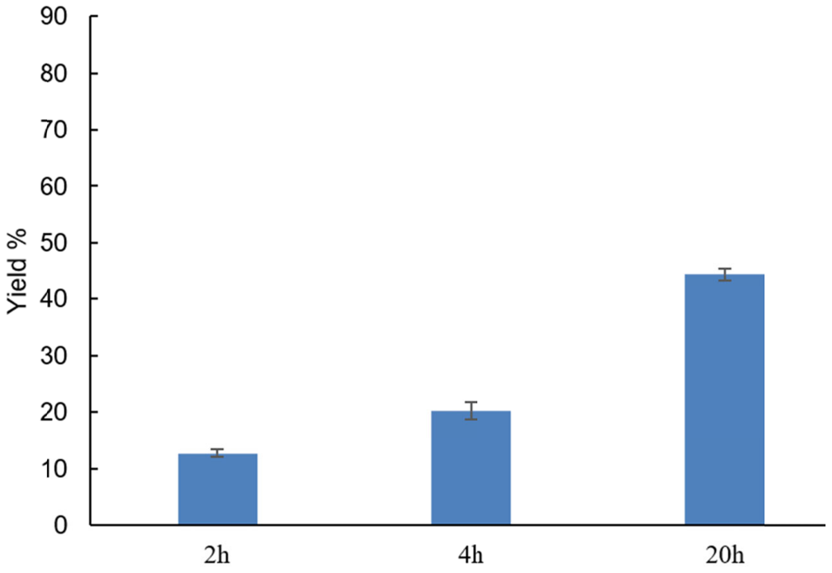

Figure S13. Hydrazone formation reaction of **1a** and **2** by integration of HMFO and LmrR_V15pAF_RMH *in vivo***.** The reaction was followed during 20 h with a final yield of 50 % of product **3a** formed.

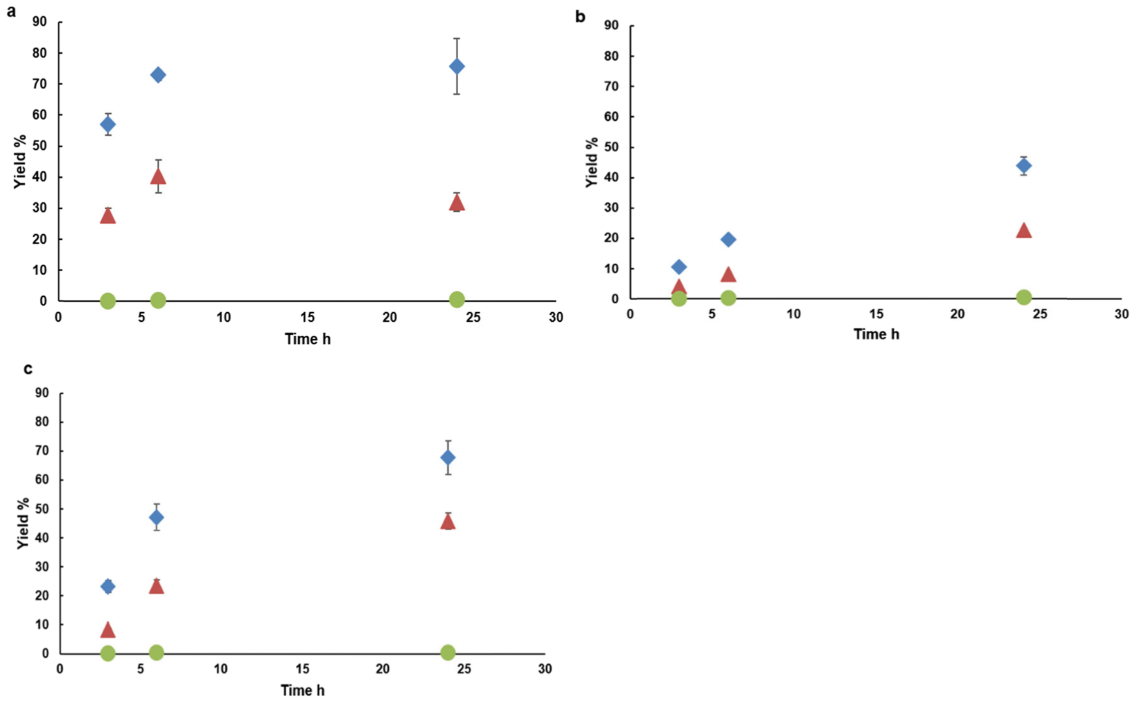

Figure S14 Hydrazone formation *in vivo* after the integration of CAR-SFP and FCS-ECH with LmrR_V15pAF_RMH in *E. coli* K12 MG1655 (RARE), using MMV at 24 ℃ with135 rpm for 24 h. (**a**) hydrazone formation using 5 mM **4b** and 50 μM **2.** CAR-SFP/LmrR_V15pAF_RMH (blue) gave 57%, 73% and 76% after 3, 6, and 24 h, respectively. Although CAR only (red) gave some background, LmrR_V15pAF_RMH only (green) did not yield any product. (**b**) 5 mM **4a** and 50 μM **2** (**c**) 5 mM **5** and 50 μM **2**, the trend was not different from **(a).** FCS-ECH_LmrRV15pAF_RMH (blue) gave 23%, 47%, and 68% of **3a** after 3, 6, and 24 h, respectively. The FCS-ECH only (red) gave a background of 46% after 24 h. The results for LmrR_V15pAF_RMH (green) were the same for all substrates (**4a, b, 5**). Error margins represent standard deviations from experiments performed in triplicate.

In vivo hydrazone formation reaction with LmrR_V15Y_RMH variant

The plasmid pET17b+ harbouring LmrR-V15Y-RMH was transformed into the *E*. coli strain K12 MG1655 (RARE). Experimental culture cells (both LmrR_V15Y_RMH and LmrR_V15pAF_RMH variants) were grown in 50 ml MMV supplemented with antibiotics at 37°C, 135 rpm until OD_{600} of 0.6. Gene expression was then induced with 1 mM isopropyl-β-d-1-thiogalactopyranoside (IPTG) incubated at 24 °C, and agitated at 135 rpm for 48 hrs. Cultures were pelleted and resuspended in phosphate buffer at pH 7, to wash away non-incorporated UAA. In the 2.5 mL cell culture in buffer was added 25 μL NBDH (2) (5 mM in DMF) solution (final concentration 50 μM) and 62.5 μL benzaldehyde (200 mM in DMF) solution (final concentration 5 mM). Then the mixture was put into the 24 °C shaker at 135 rpm for 3 hours. The formation of the product was determined by HPLC. Take 500 μL reaction culture into 2 ml Eppendorf, added 100 μL IS (1 mM 2-phenylquinoline in DMF), then extracted with 1ml ethyl acetate, after the evaporation, the residue was

resuspended in 1200 µL CH3CN. 200 µL of this solution was placed into HPLC vial and the sample was analysed by HPLC.

The hydrazone product with LmrR_V15pAF_RMH variant had a higher yield of 86 % compared to that of LmrR_V15Y_RMH variant which gave a yield of 39 %, indicating that the pAF can catalyse the reaction better than tyrosine. This was done to proof that our protein had pAF incorporated and not tyrosine.

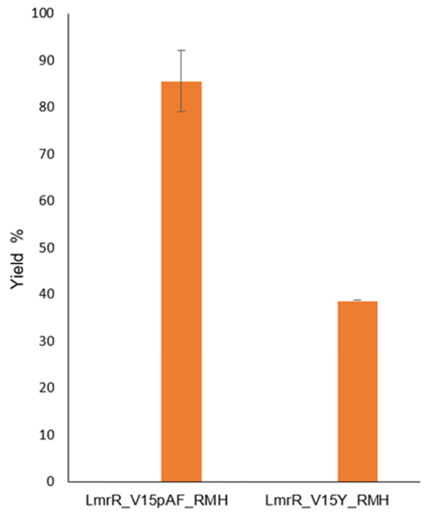

Figure S15. Yield **3b** of hydrazone product using phosphate buffer in E.coli K12 MG1655 (RARE). Conditions: 5 mM **1b** and 50 µM **2** in phosphate buffer pH 7, at 24 ℃/ 135 rpm for 3 h. Error margins represent standard deviations from the average of duplicates.

Background reaction of hydrazone formation

In finding out the main contributor to the background reaction in MMV, we decided to the reaction in MMV and buffer whereby the presence of some vitamins are increased (mainly aromatic amines) in some samples and limited in other samples to see the effect. Here, we set 3 different types of control reactions with 5 mM benzaldehyde and 50 µM NBDH final concentration. The reaction was set up in phosphate buffer at pH 7, MVV media (with 2 mM Folic acid, 4-amino benzoic acid and thiamine x HCL) and MMV media (without p-aminobenzoic acid) and incubated at 24 ℃/135 rpm for 2 h. 500 ul reaction mix was taken at the beginning and at the end of the reaction for extraction, product was extracted with 0.9 ml ethyl acetate and 0.1 ml of 1 mM internal.and was then detected with the HPLC.

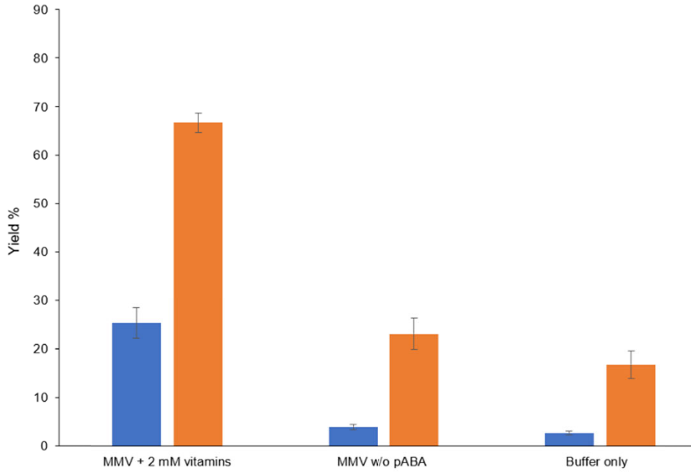

Figure S16. Yield of hydrazone product formed in control samples for 10 mins (blue) and 2 h (orange). Samples with MMV and 2 mM vitamins (folic acid, thiamine and 4-amino benzoic acid) had a higher product detection, both at the beginning and at the end of the reaction (25% and 67% respectively). Meanwhile that of MMV without 4-amino benzoic acid and buffer had lower yields of 4% and 3% after 10 mins and 23% and 17% after 2 h. This clearly shows that the background reaction is caused by the presence of aromatic amines in MMV.

HPLC Chromatogram for hydrazone

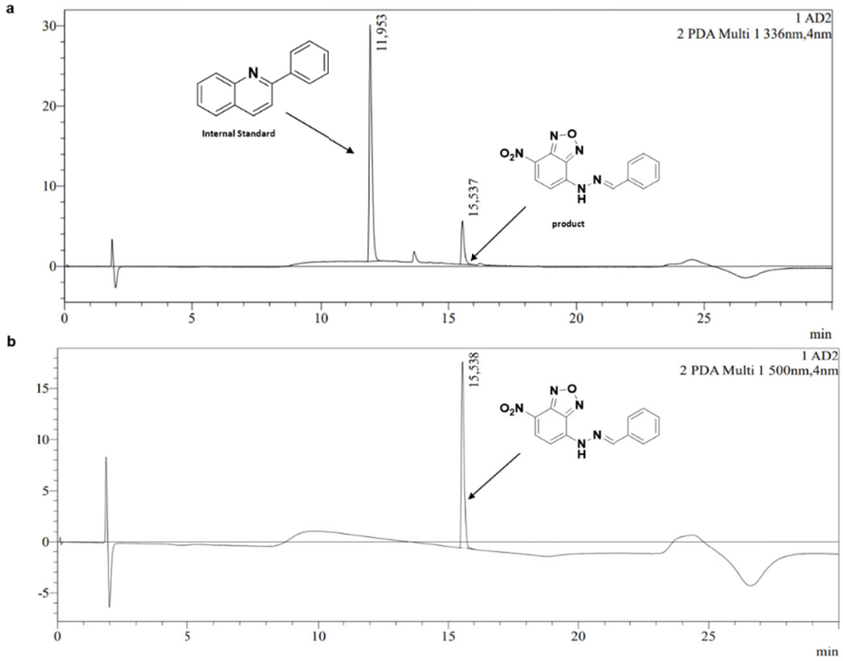

Figure S17. HPLC chromatogram of Hydrazone product **3b** formed by integration of HMFO and LmrR_V15pAF_RMH *in vivo*. (**a**) At wavelength 336 nm. (**b**) At wavelength 500 nm.

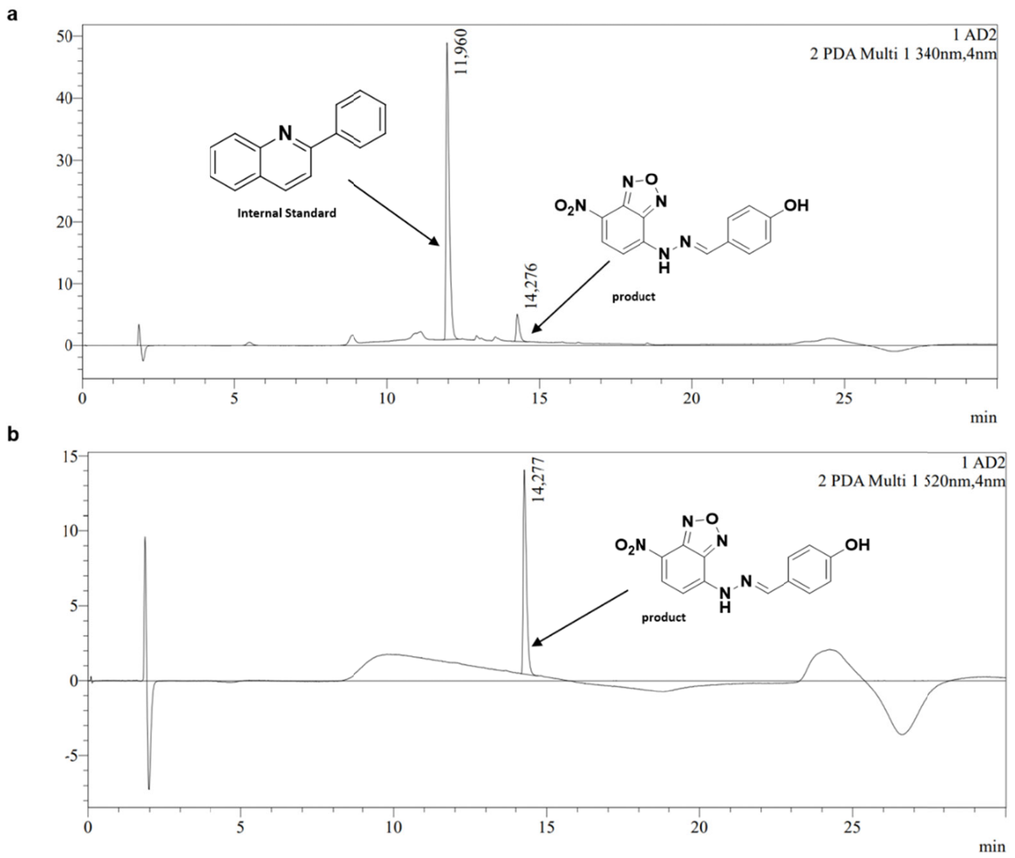

Figure S18. HPLC chromatogram of Hydrazone product **3b** in the presence only HMFO *in vivo* (i.e. in absence of LmrR_V15pAF_RMH). (**a**) At wavelength 336 nm. (**b**) At wavelength is 500 nm.

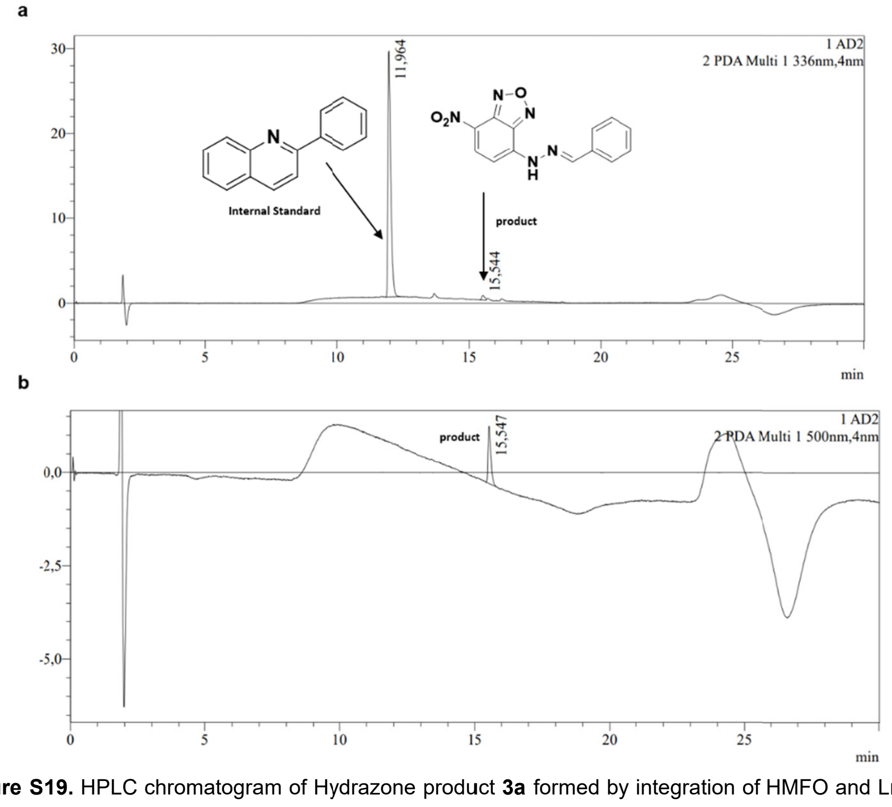

Figure S19. HPLC chromatogram of Hydrazone product **3a** formed by integration of HMFO and LmrR_V15pAF_RMH *in vivo*. (**a**) At wavelength 340 nm. (**b**) At wavelength is 520 nm.

Reductive amination using $NaCNBH_3$

Generation of iminium intermediate by the condensation of aldehydes, in this case benzaldehyde and pAF, is done by reductive amination using $NaCNBH_3$. Here we showed that pAF was incorporated into LmrR_V15TAG_RMH and not tyrosine by performing reductive amination on both variants. The reductive amination reactions were conducted with 50 µM LmrR_V15pAF and 1 mM benzaldehyde in 300 µL scale stirring for 2 hours at 4 °C. After that, 100 µL of a 90 mM NaCNBH3 solution in water was added and the reaction was stirred for 1 hour. The reaction was then analysed by LCMS.

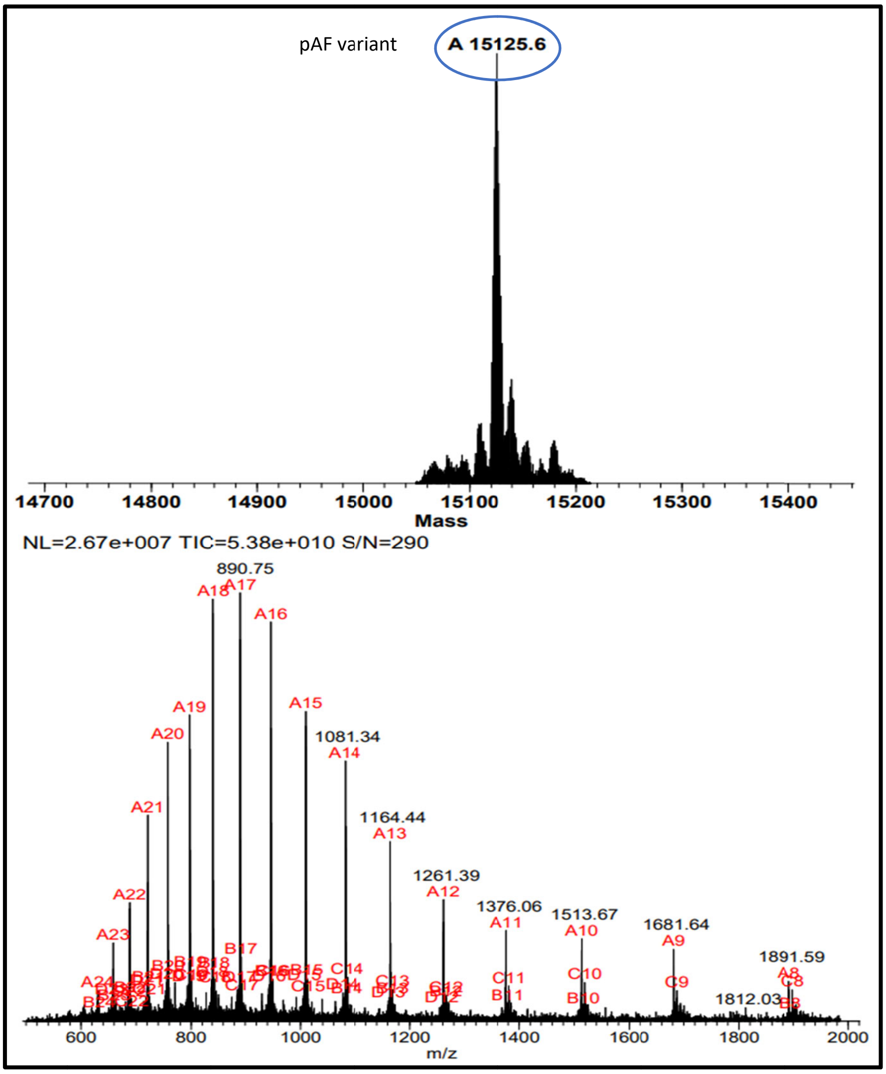

Mass before reductive amination, LmrR_V15pAF_RMH variant.

Expected mass = 15126: observed mass = 15125.6

Mass after reductive amination, LmrR_V15pAF_RMH variant

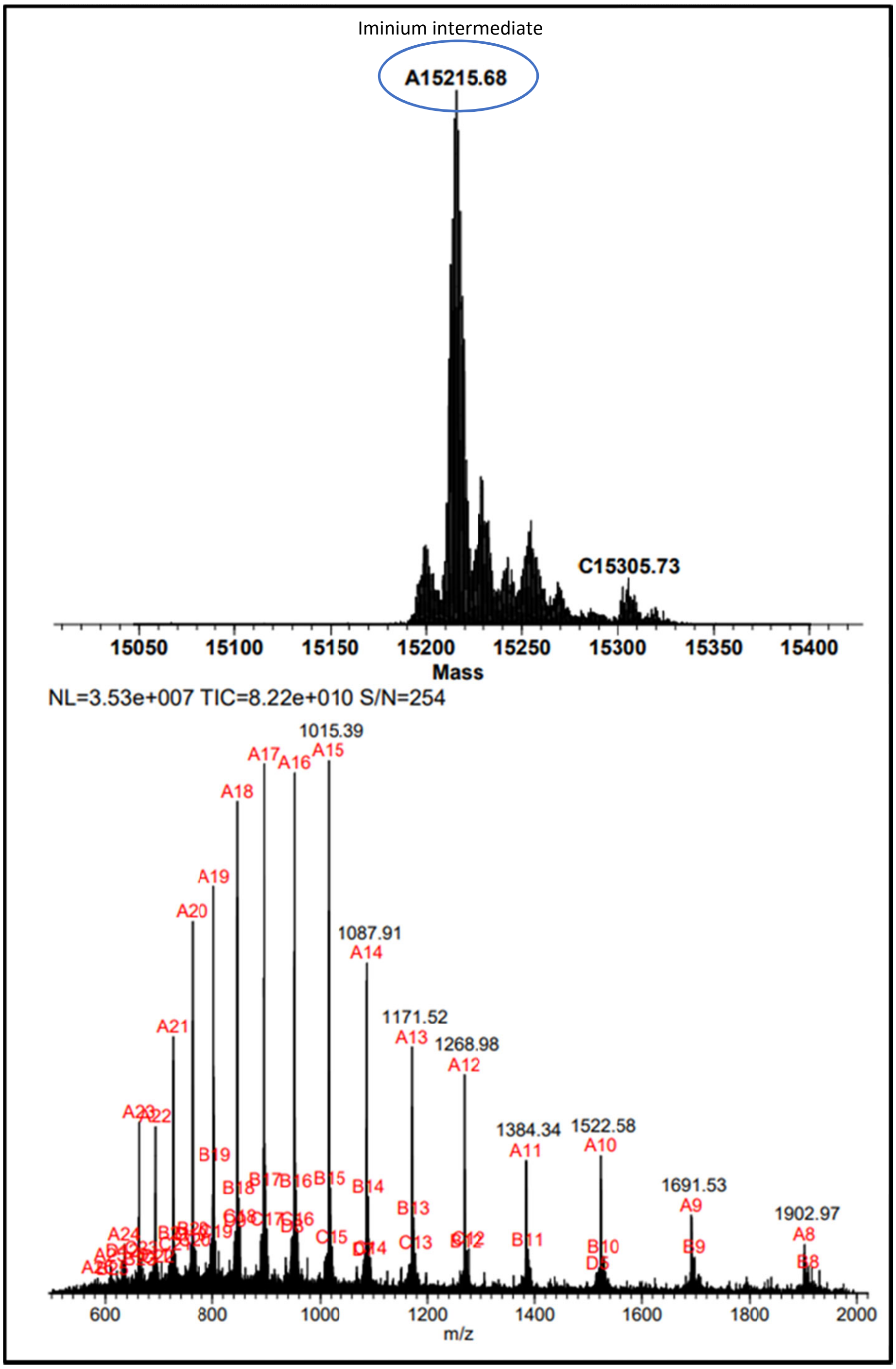

Expected mass = 15216: observed mass = 15215.68

Mass before reductive amination, LmrR_V15Y_RMH variant

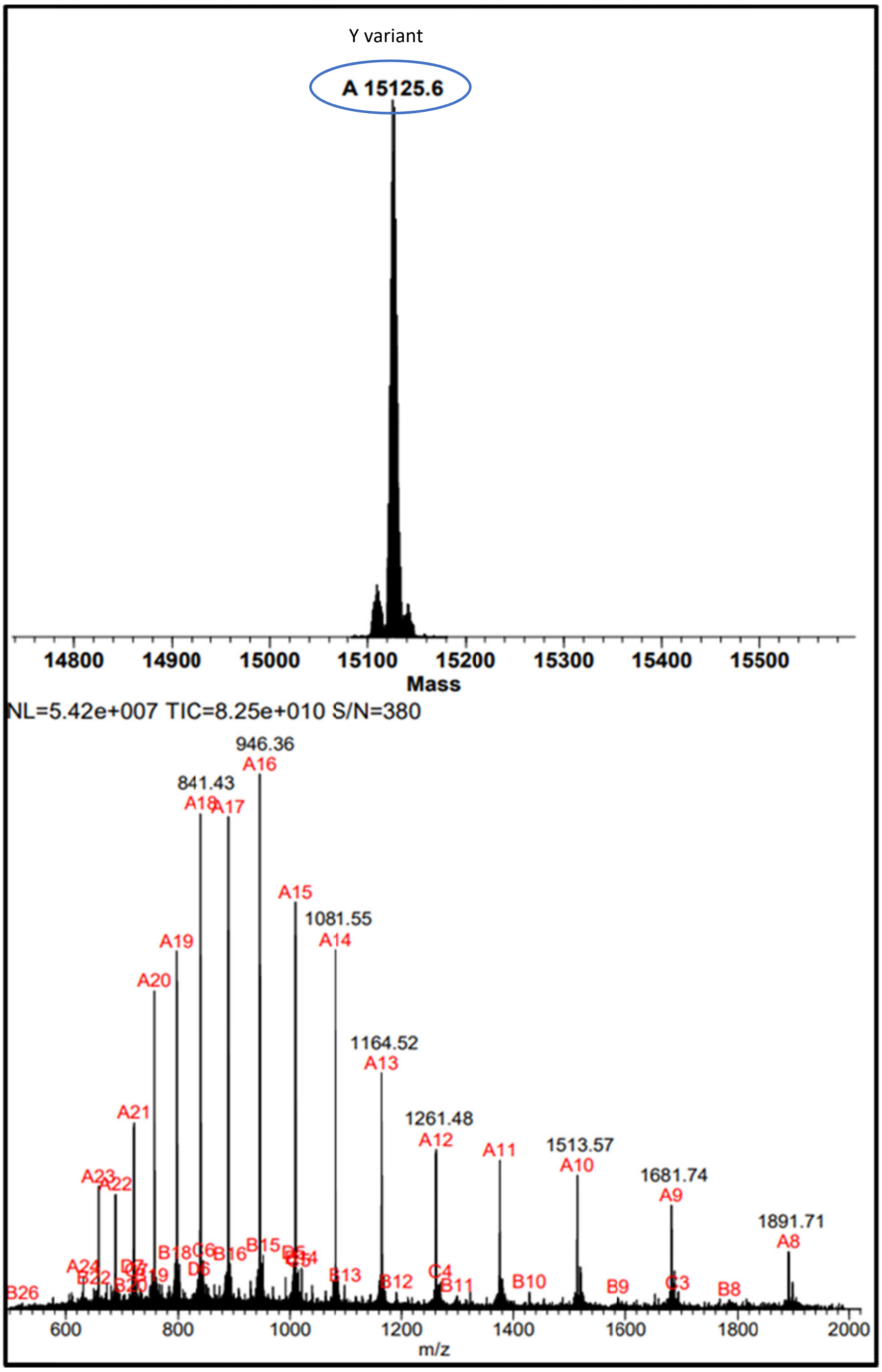

Expected mass = 15127; observed mass = 15125.6

Mass after reductive amination, LmrR_V15Y_RMH variant

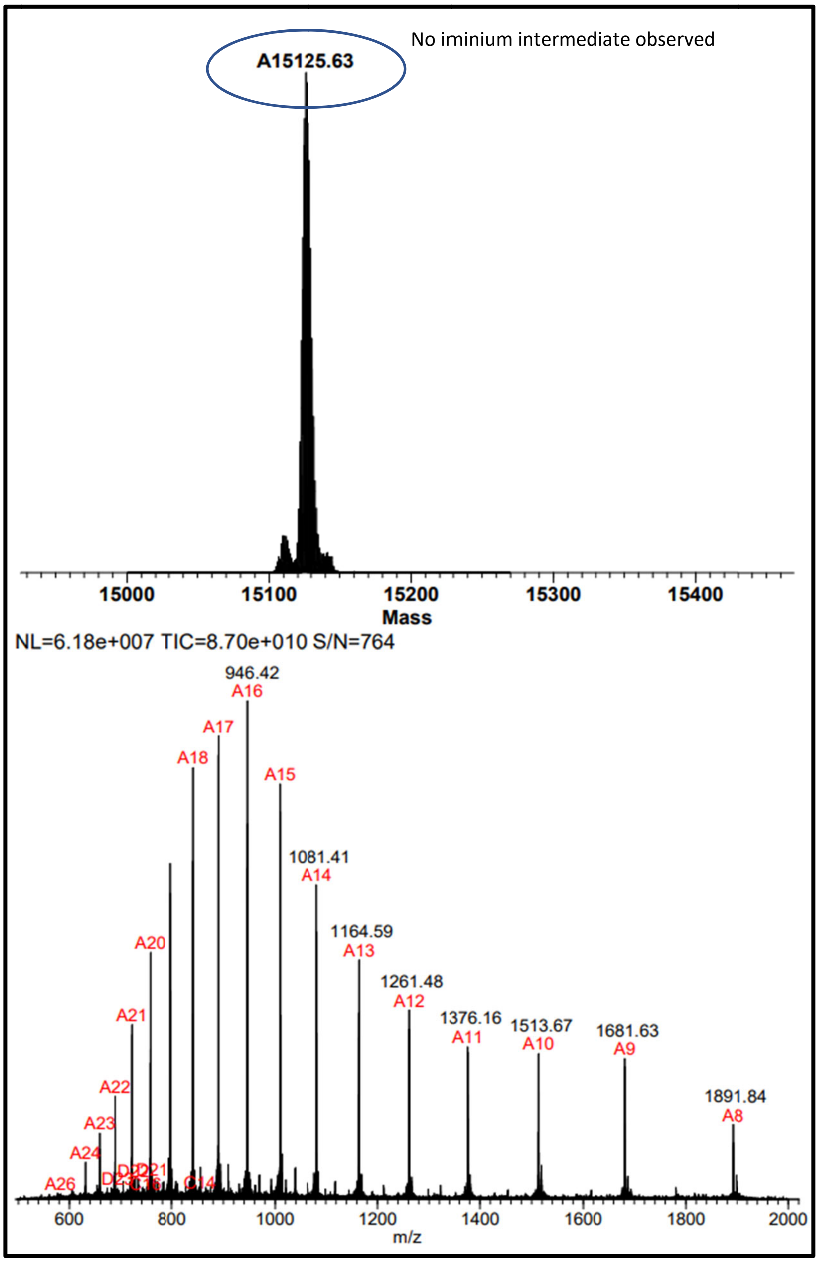

Expected mass = 15127; observed mass = 15125.63

Figure S20. Mass spectrometry for LmrR_V15pAF_RMH and LmrR_V15Y_RMH before and after trapping of the transiently formed iminium ion in the presence of benzaldehyde and $NaCNBH_3$. According the mass spectrometry, the mass of the LmrR_V15pAF_RMH variant after reductive amination corresponded to the expected mass of the iminium ion formed with pAF in the presence benzaldehyde and $NaCNBH_3$. On the other hand, there was no iminium intermediate formed with the LmrR_V15Y_RMH as expected. This confirmed our argument that, pAF was successfully incorporated into LmrR_V15X_RMH.

Recycling Experiment

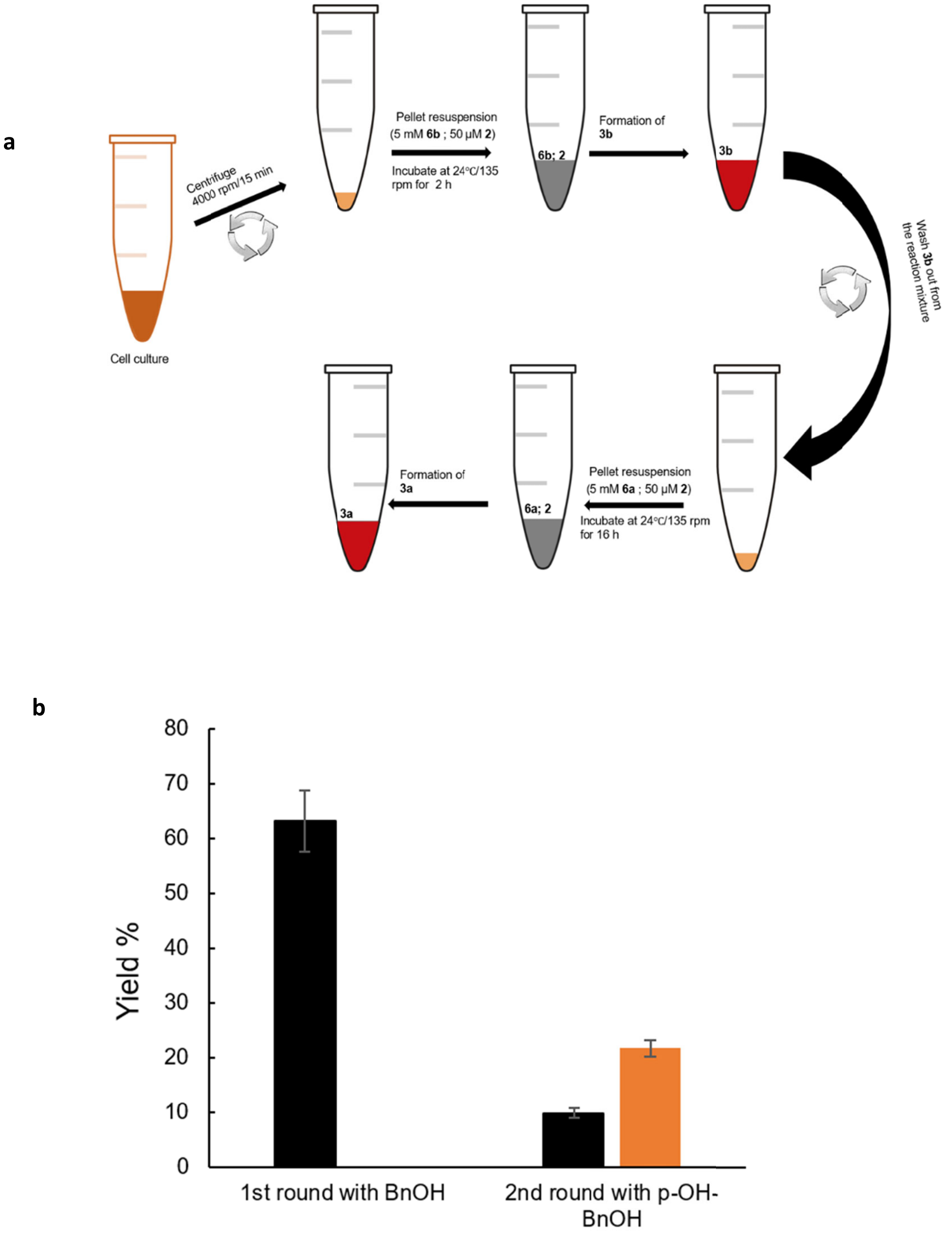

Figure S21. Recycling experiment to establish that catalysis takes place in the cell and the cell remains intact during and after catalysis (**a**) Schematic workflow of an *in vivo* recycling experiment of hydrazone formation reaction using HMFO/LmrR_V15pAF _RMH. Products (**3a,b**) were analyzed with the HPLC after extraction. (**b**) Product yields detected after each catalysis experiment, first round of reaction resulted in 63 % of **3b** (black) and the second round yielded 22 % of **3a** (orange) with 10 % residual **3a**.

Cell viability test after catalysis

After the *in vivo* catalysis, reaction mixtures were spun down at 8000 rpm for 30 mins and resuspended in LB medium. Serial dilutions (10x - 10^{-8}) of selected samples were plated on LB plates containing 100 µg/mL ampicillin, 10 µg/mL spectinomycin and 50 µg/mL Kanamycin for 3 plasmid system and plates containing 100 µg/mL ampicillin and 10 µg/mL spectinomycin for 2 plasmid system. Plates were then incubated at 37 °C overnight. The following day, colonies were counted, plates with 10x diluted were confluent and therefore not countable. However plates with dilutions from 10^{-5} were countable.

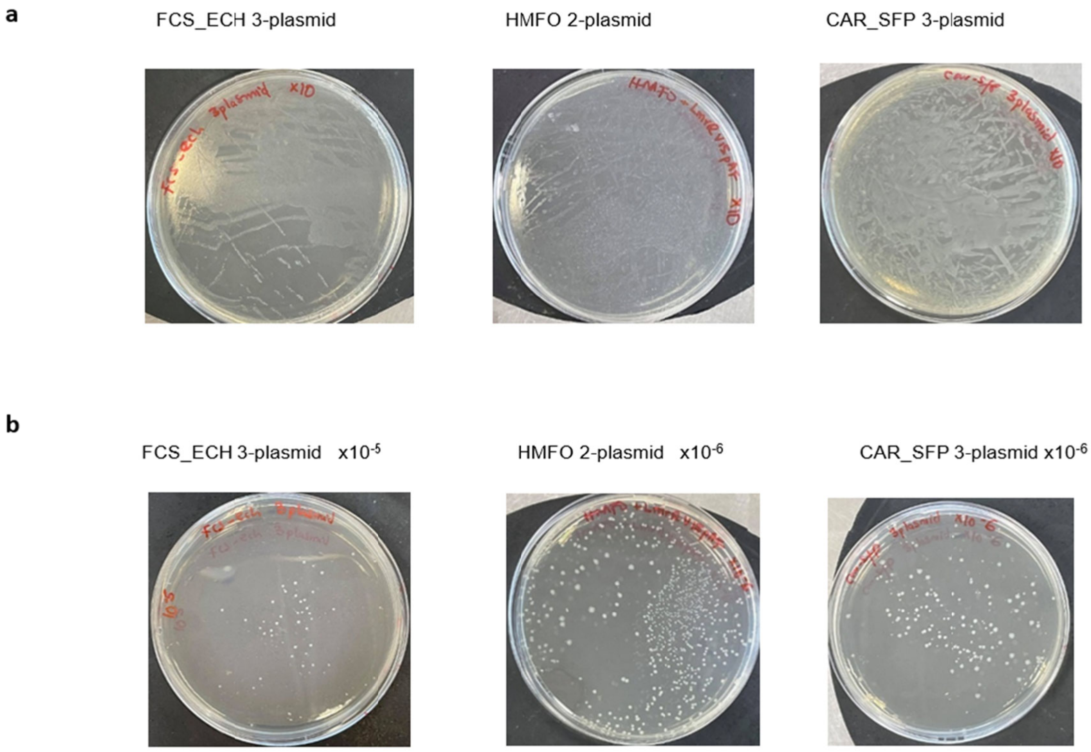

Figure S22. Cell viability test after hydrazone formation reaction. (**a**) Image of the confluency of colonies after 10 times dilution. (**b**) Plate with FCS-ECH 3-plasmid had 45 colonies, HMFO 2-plasmid had 336 colonies and CAR-SFP 3-plasmid had 141 colonies after several serial dilutions.

After the viability test, LmrR_V15pAF_RMH/HMFO system was used to repeat the cell viability test and this time with 2 different substrates. After protein expression at 24℃/135 rpm for 48 hours, cultures were collected and spun down at 4000 rpm for 10 mins. The pellet was resuspended in 50 mM potassium phosphate buffer at pH 6.5, reaction was

performed by adding 5 mM of benzylalcohol and 50 μM of NBD-H to the cell and incubated at 24℃/135 rpm for 2 hours. The reaction mixtures were then spun down and the cells resuspended in LB media, serial dilutions were done down to 10^{-6} and 200 μl was plated on LB agar each dilution and incubated at 37℃ overnight. A single colony from the 10^{-6} dilution plate was picked and grown in LB media while expressing LmrR_V15pAF_RMH/HMFO. Catalysis was performed as previously described with the new cells, now using 5 mM p-hydroxybenzyl alcohol and 50 μM NBDH as substrates in 50 mM potassium phosphate buffer at pH 6.5. After overnight reaction at 24℃/135 rpm, the samples were again spun down, resuspended in LB and serial dilutions down to 10^{-9} were plated on agar. Colonies on the plates with 10^{-8} and 10^{-9} dilutions yielded 419 and 60 colonies respectively.

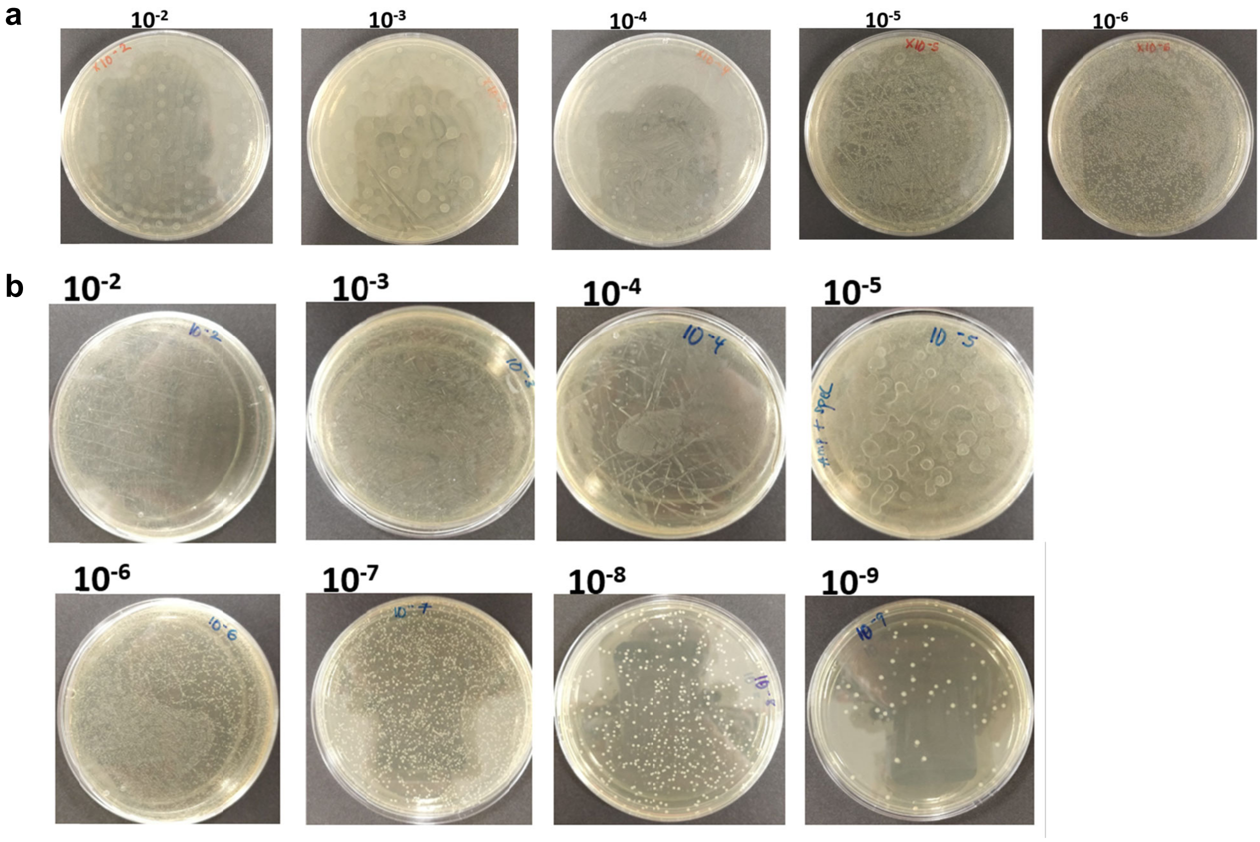

Figure S23. Agar plates with colonies after viability test with LmrR_V15pAF_RMH/HMFO system (**a**) images of colony plates after the first round of catalysis with 5 mM of benzylalcohol and 50 μM of NBD-H for 2 hours. (**b**)Colony plates after second round of catalysis 5 mM p-hydroxybenzyl alcohol and 50 uM NBDH for overnight. Colonies counted on the 10^{-8} and 10^{-9} plates were estimated to be 6×10^{7} cells/ml for plate 10^{-8} and 2×10^{7} cells/ml for plate 10^{-9} viable cells after catalysis, compared to 1.92×10^{10} cells/ml at the onset.

Gene sequence used in the work

LmrR-V15TAG RMH

ATGGGTGCCGAAATCCCGAAAGAAATGCTGCGTGCTCAAACCAATTAGATCCTGCTGATGGTCCTGAAACAA
GGCGATAACTATGTGTATGGCATTATCAAACAGGTGAAAGAAGCGAGCAACGGTGAAATGGAACTGAATGAA
GCCACCCTGTATACGATTTTTGATCGTCTGGAACAGGACGGCATTATCAGCTCTTACTGGGGTGATGAAAGT
CAAGGCGGTCGTCGCAAATATTACCGTCTGACCGAAATCGGCCATGAAAACATGCGCCTGCGGCATGAATC
CTGGAGTCGTGTGGACAAAATCATTGAAAATCTGGAAGCAAACAAAAAATCTGAAGCGATCAAATCTAGAGG
TGGCAGCGGTGGCTGGAGCCACCCGCAGTTCGAAAAATAA

HMFO

ATGACTGATACGATTTTTGACTACGTGATTGTTGGCGGTGGCACGGCGGGTAGCGTTCTGGCCAACCGTCT
GTCCGCCCGTCCGGAGAATCGCGTGTTGCTGATTGAGGCCGGTATTGATACCCCGGAAAACAATATTCCGC
CGGAGATCCACGATGGCCTGCGCCCGTGGCTGCCGCGTCTGAGCGGTGATAAGTTCTTTTGGCCGAATCT
GACCGTCTACCGTGCCGCGGAACACCCGGGTATCACGCGCGAGCCGCAGTTCTATGAACAAGGCCGTCTG
CTGGGCGGTGGTAGCAGCGTGAACATGGTCGTTTCTAACCGTGGTCTGCCTCGCGACTATGACGAATGGCA
GGCACTGGGCGCAGATGGTTGGGATTGGCAGGGTGTTCTGCCGTACTTCATCAAGACCGAGCGTGACGCG
GACTACGGTGACGACCCGTTGCATGGCAATGCGGGTCCGATTCCGATCGGTCGCGTCGATTCGCGTCACT
GGAGCGACTTCACGGTGGCGGCAACCCAAGCTCTGGAAGCGGCTGGCCTGCCGAACATTCACGACCAAAA
CGCACGTTTTGATGACGGTTACTTCCCACCGGCATTTACGTTGAAAGGTGAAGAGCGCTTCAGCGCCGCAC
GCGGTTATCTGGATGCGAGCGTCCGTGTGCGTCCGAACCTGAGCCTGTGGACTGAGAGCCGTGTCCTGAA
GCTGCTGACCACTGGCAATGCAATCACCGGTGTGAGCGTGCTGCGTGGTCGCGAAACCCTGCAAGTTCAAG
CGCGCGAGGTCATCCTGACCGCCGGTGCGTTGCAAAGCCCAGCGATTCTGTTGCGCACCGGCATCGGCCC
TGCGGCGGATCTGCACGCACTGGGTATTCCTGTTCTGGCAGACCGTCCGGGTGTTGGTCGCAATCTGTGG
GAGCACAGCTCTATCGGTGTGGTTGCCCCGCTGACCGAGCAGGCACGTGCAGACGCCAGCACGGGTAAAG
CCGGCTCTCGCCATCAACTGGGTATCCGTGCGTCGTCCGGCGTAGATCCGGCGACGCCTAGCGACCTGTT
TCTGCATATCCATGCTGATCCAGTCAGCGGTCTGGCAAGCGCTCTGTTCTGGGTGAACAAGCCAAGCTCCA
CCGGCTGGCTGAAGCTGAAGGACGCGGACCCGTTTAGCTACCCGGACGTAGACTTCAATCTGCTGAGCGAT
CCGCGCGACTTGGGTCGTCTGAAAGCGGGCCTGCGTCTGATCAAACATTACTTCGCATATCCGTCCCTGGC
GAAATATGGTTTGGCGCTGGCATTGAGCCGTTTTGAGGCACCGCAGCCGGGTGGTCCGCTGCTGAACGAC
CTGTTGCAGGACGAAGCCGCCCTGGAACGCTATTTGCGTACGAACGTCGGCGGTGTTTGGCATGCGAGCG
GCACGGCGCGTATCGGCCGTGCGGATGATTCCCAGGCTGTTGTCGATAAAGCGGGTCGTGTGTACGGCGT
CACCGGCCTGCGTGTTGCGGACGCAAGCATTATGCCGACCGTTCCGACCGCCAATACCAATCTGCCGACG
CTGATGCTGGCTGAGAAAATTGCGGATGCGATTCTGACCCAGGCTTAA

CAR-SFP

<u>CCATGG</u>GCAGCAGCCATCACCATCATCACCACAGCCAGGATCCGAATTCGATGGCTGTGGACTCGCCGGAT

GAACGCCTGCAACGCCGTATCGCCCAACTGTTTGCCGAAGATGAACAAGTGAAAGCTGCCCGCCCGCTGGA

AGCAGTTAGCGCGGCCGTCTCTGCACCGGGTATGCGTCTGGCTCAGATCGCAGCTACGGTGATGGCTGGT

TATGCGGATCGTCCGGCGGCGGGCCAGCGTGCTTTCGAACTGAATACCGATGACGCAACCGGCCGTACCA

GCCTGCGTCTGCTGCCGCGTTTTGAAACCATTACGTACCGCGAACTGTGGCAGCGTGTCGGCGAAGTGGCA

GCTGCGTGGCATCACGACCCGGAAAACCCGCTGCGTGCGGGTGATTTTGTGGCCCTGCTGGGCTTCACCA

GCATTGATTATGCAACGCTGGATCTGGCTGACATCCATCTGGGTGCGGTTACCGTGCCGCTGCAAGCGAGC

GCGGCGGTGTCCCAACTGATTGCAATCCTGACCGAAACGAGTCCGCGCCTGCTGGCGTCCACCCCGGAAC

ATCTGGATGCTGCGGTGGAATGCCTGCTGGCAGGCACCACGCCGGAACGTCTGGTGGTTTTCGATTATCAC

CCGGAAGATGACGATCAGCGCGCCGCATTTGAAAGTGCGCGTCGCCGTCTGGCAGATGCAGGTTCCCTGG

TGATCGTTGAAACCCTGGACGCGGTGCGTGCGCGTGGCCGTGATCTGCCGGCTGCGCCGCTGTTTGTCCC

GGATACCGACGATGACCCGCTGGCGCTGCTGATTTATACGTCAGGTTCGACCGGCACGCCGAAAGGTGCC

ATGTACACCAATCGTCTGGCCGCAACGATGTGGCAGGGCAACTCAATGCTGCAAGGCAACAGCCAACGCGT

TGGCATTAACCTGAATTATATGCCGATGAGTCATATTGCGGGTCGTATCTCCCTGTTCGGCGTGCTGGCGCG

TGGCGGCACCGCATACTTTGCTGCGAAATCAGACATGAGCACCCTGTTTGAAGATATTGGCCTGGTTCGCC
CGACCGAAATCTTTTTCGTTCCGCGTGTCTGTGACATGGTGTTTCAGCGCTATCAAAGCGAACTGGATCGCC
GTTCTGTCGCTGGTGCGGATCTGGACACCCTGGACCGCGAAGTGAAAGCGGATCTGCGTCAGAATTACCTG
GGCGGTCGCTTCCTGGTTGCAGTCGTGGGCTCGGCTCCGCTGGCCGCAGAAATGAAAACGTTTATGGAAA
GCGTGCTGGACCTGCCGCTGCATGATGGTTATGGCAGTACCGAAGCCGGCGCATCCGTTCTGCTGGATAAC
CAGATCCAACGTCCGCCGGTCCTGGACTATAAACTGGTCGATGTGCCGGAACTGGGTTACTTTCGCACGGA
TCGTCCGCACCCGCGTGGCGAACTGCTGCTGAAAGCAGAAACCACGATTCCGGGTTATTACAAACGCCCGG
AAGTTACGGCGGAAATCTTTGATGAAGACGGCTTCTATAAAACCGGCGATATTGTGGCCGAACTGGAACATG
ACCGCCTGGTTTACGTGGATCGTCGTAACAATGTTCTGAAACTGTCCCAGGGCGAATTTGTGACCGTTGCGC
ACCTGGAAGCTGTGTTCGCGAGCAGCCCGCTGATCCGTCAAATTTTTATCTATGGTAGTTCCGAACGCAGTT
ACCTGCTGGCCGTCATTGTGCCGACCGATGACGCACTGCGTGGCCGCGATACCGCTACGCTGAAAAGCGC
TCTGGCGGAATCTATTCAGCGTATCGCCAAAGACGCAAATCTGCAACCGTATGAAATTCCGCGCGATTTTCT
GATCGAAACCGAACCGTTCACGATTGCCAATGGCCTGCTGAGCGGTATCGCAAAACTGCTGCGCCCGAACC
TGAAAGAACGTTATGGTGCGCAGCTGGAACAAATGTACACCGACCTGGCTACGGGCCAGGCAGATGAACTG
CTGGCCCTGCGCCGTGAAGCTGCGGATCTGCCGGTGCTGGAAACCGTTAGCCGTGCCGCAAAAGCGATGC
TGGGTGTGGCAAGCGCGGATATGCGTCCGGACGCACATTTTACCGATCTGGGCGGTGACAGCCTGTCTGC
ACTGAGTTTTTCCAACCTGCTGCACGAAATCTTCGGTGTTGAAGTCCCGGTGGGTGTTGTCGTGTCTCCGGC
AAACGAACTGCGTGATCTGGCGAATTATATTGAAGCCGAACGCAACAGTGGCGCAAAACGTCCGACCTTCA
CGTCAGTGCATGGCGGTGGCTCGGAAATTCGTGCTGCGGATCTGACCCTGGACAAATTTATCGATGCACGC
ACGCTGGCCGCAGCTGATTCTATTCCGCACGCCCCGGTGCCGGCACAGACCGTTCTGCTGACGGGTGCGA
ATGGCTATCTGGGTCGTTTCCTGTGCCTGGAATGGCTGGAACGCCTGGATAAAACCGGCGGCACCCTGATT
TGTGTTGTCCGTGGTAGCGACGCGGCGGCGGCACGTAAACGTCTGGATTCAGCCTTTGATAGCGGCGATC
CGGGCCTGCTGGAACATTATCAGCAACTGGCAGCACGTACCCTGGAAGTGCTGGCAGGCGATATTGGTGAC
CCGAACCTGGGCCTGGATGACGCGACCTGGCAGCGTCTGGCAGAAACGGTCGATCTGATTGTGCATCCGG
CAGCTCTGGTGAATCACGTTCTGCCGTACACCCAGCTGTTTGGCCCGAACGTGGTTGGCACCGCGGAAATT
GTGCGCCTGGCTATCACCGCGCGTCGTAAACCAGTGACCTATCTGTCTACGGTTGGCGTCGCAGATCAGGT
TGACCCGGCTGAATACCAAGAAGATAGCGATGTGCGTGAAATGTCTGCGGTGCGTGTCGTGCGCGAAAGCT
ATGCCAACGGTTACGGCAATTCTAAATGGGCTGGTGAAGTGCTGCTGCGCGAAGCGCATGATCTGTGCGGT
CTGCCGGTGGCAGTTTTTCGTTCAGATATGATTCTGGCACACTCGCGCTATGCTGGTCAGCTGAATGTCCAA
GATGTGTTCACCCGTCTGATTCTGTCACTGGTTGCTACGGGCATCGCGCCGTATTCGTTTTACCGCACCGAT
GCAGACGGTAACCGTCAGCGCGCCCATTACGATGGTCTGCCGGCAGATTTCACCGCGGCGGCGATTACGG
CGCTGGGTATCCAGGCCACCGAAGGCTTTCGCACGTATGATGTGCTGAATCCGTATGATGACGGTATTAGT
CTGGACGAATTTGTTGATTGGCTGGTCGAATCCGGCCATCCGATTCAGCGTATCACGGATTATTCAGACTGG
TTTCACCGCTTCGAAACCGCCATCCGTGCACTGCCGGAAAAACAGCGTCAAGCCAGCGTGCTGCCGCTGCT
GGATGCATACCGTAACCCGTGTCCGGCCGTTCGCGGTGCAATTCTGCCGGCTAAAGAATTTCAGGCTGCGG
TCCAAACCGCGAAAATTGGCCCGGAACAGGATATTCCGCACCTGAGTGCCCCGCTGATTGATAAATACGTG
TCTGACCTGGAACTGCTGCAACTGCTGGGTAGTGGCTCTGGACTGGTGGGTGCCCTGATGCACGTGATGCA
GAAGCGCAGCCGCGCCATCCACTCCTCCGACGAAGGGGAGGACCAGGCTGGCGATGAAGATGAAGATTGA
GCGGCCGC

FSC

GGATCCGGTGCGTAACCAGGGTCTGGGTTCTTGGCCGGTTCGTCGTGCTCGTATGTCTCCGCACGCTACCG
CTGTTCGTCACGGTGGTACCGCTCTGACCTACGCTGAACTGTCTCGTCGTGTTGCTCGTCTGGCTCACGGT
CTGCGTGAAGCTGGTGTTCGTCCGGGTGACCGTGTTGCTTACCTGGGTCCGAACCACCCGGCTTACCTGGA
AACCCTGTTCGCTTGCGGTCAGGCTGGTGCTGTTTTCGTTCCGCTGAACTTCCGTCTGGGTGTTCCGGAACT

GGACCACGCTCTGGCTGACTCTGGTGCTTCTGTTCTGATCCACACCCCGGAACACGCTGAAACCGTTGCTG
CTCTGGCTGGTGACCGTCTGCTGCGTGTTCCGGCTGGTGAACTGGAAGCTGCTGACGACGAACCGCTGGA
CCTGCCGGTTGGTCTGGACGACGTTTGCCTGCTGATGTACACCTCTGGTTCTACCGGTCGTCCGAAAGGTG
CTATGCTGACCCACGGTAACCTGACCTGGAACTGCGTTAACGTTCTGGTTGAAACCGACCTGGCTTCTGAC
GAACGTGCTCTGGTTGCTGCTCCGCTGTTCCACGCTGCTGCTCTGGGTATGGTTTGCCTGCCGACCCTGCT
GAAAGGTGGTACCGTTATCCTGCACTCTGCTTTCGACCCGGGTGCTGTTCTGTCTGCTGTTGAACAGGAAC
GTGTTACCCTGGTTTTCGGTGTTCCGACCATGTACCAGGCTATCGCTGCTCACCCGCGTTGGCGTTCTACC
GACCTGTCTTCTCTGCGTACCCTGCTGTGCGGTGGTGCTCCGGTTCCGGCTGACCTGGCTGGTCGTTACCT
GGACCGTGGCCTGGCGTTCGTTCAGGGTTACGGTATGACCGAAGCTGCTCCGGGTGTTCTGGTTCTGGAC
CGTGCTCACGTTGCTGAAAAAATCGGTTCTGCTGGTGTTCCGTCTTTCTTCACCGACGTTCGTGTTGCTGGT
CCGTCTGGTGAACCGGTTCCGCCGGGTGAAAAAGGTGAAATCGTTGTTTCTGGTCCGAACGTTATGAAAGG
TTACTGGGGTCGTCCGGAAGCTACCGCTGAAGTTCTGCGTGACGGTTGGTTCCGTTCTGGTGACGTTGCTA
CCGTTGACGGTGACGGTTACTTCCACGTTGTTGACCGTCTGAAAGACATGATCATCTCTGGTGGTGAAAACA
TCTACCCGGCTGAAGTTGAAAACGAACTGTACGGTTACCCGGGTGTTGAAGCTTGCGCTGTTATCGGTGTTC
CGGACCCGCGTTGGGGTGAAGTTGGTAAAGCTGTTGTTGTTCCGGCTGCTGGTTCTCGTATCGACGGTGCT
GAACTGCTGGCTTGGCTGCGTACCCGTCTGGCTGGTTACAAAGTTCCGAAATCTGTTGAATTCACCGACCGT
CTGCCGACCACCGGTTCTGGTAAAATCCTGAAAGGTGAAGTTCGTCGTCGTTTCGGTTAAGCGGCCGC

ECH

CATATGTCTACCGCTGTTGGTAACGGTCGTGTTCGTACCGAACCGTGGGGTGAAACCGTTCTGGTTGAATTC
GACGAAGGTATCGCTTGGGTTACCCTGAACCGTCCGGACAAACGTAACGCTATGAACCCGACCCTGAACGA
CGAAATGGTTCGTGTTCTGGACCACCTGGAAGGTGACGACCGTTGCCGTGTTCTGGTTCTGACCGGTGCTG
GTGAATCTTTCTCTGCTGGTATGGACCTGAAAGAATACTTCCGTGAAGTTGACGCTACCGGTTCTACCGCTG
TTCAGATCAAAGTTCGTCGTGCTTCTGCTGAATGGCAGTGGAAACGTCTGGCTAACTGGTCTAAACCGACCA
TCGCTATGGTTAACGGTTGGTGCTTCGGTGGTGCTTTCACCCCGCTGGTTGCTTGTGACCTGGCGTTCGCT
GACGAAGACGCTCAGTTCGGTCTGTCTGAAGTTAACTGGGGTATCCCACCGGGTGGCGTTGTAAGCCGTGC
TCTGGCTGCTACCGTTCCGCAGCGTGACGCTCTGTACTACATCATGACCGGTGAACCGTTCGACGGTCGTC
GTGCTGCTGAAATGCGTCTGGTTAACGAAGCTCTGCCGGCTGACCGTCTGCGTGAACGTACCCGTGAAGTT
GCTCTGAAACTGGCTTCTATGAACCAGGTTGTTCTGCACGCTGCTAAAACCGGTTACAAAATCGCTCAGGAA
ATGCCGTGGGAACAGGCTGAAGACTACCTGTACGCTAAACTGGACCAGTCTCAGTTCGCTGACAAAGCTGG
TGCTCGTGCTAAAGGTCTGACCCAGTTCCTGGACCAGAAATCTTACCGTCCGGGTCTGTCTGCTTTCGACCC
GGAAAAATAACTCGAG

Note

All underlined sequence are the restriction sites used for cloning

Plasmid maps of plasmids used in this study

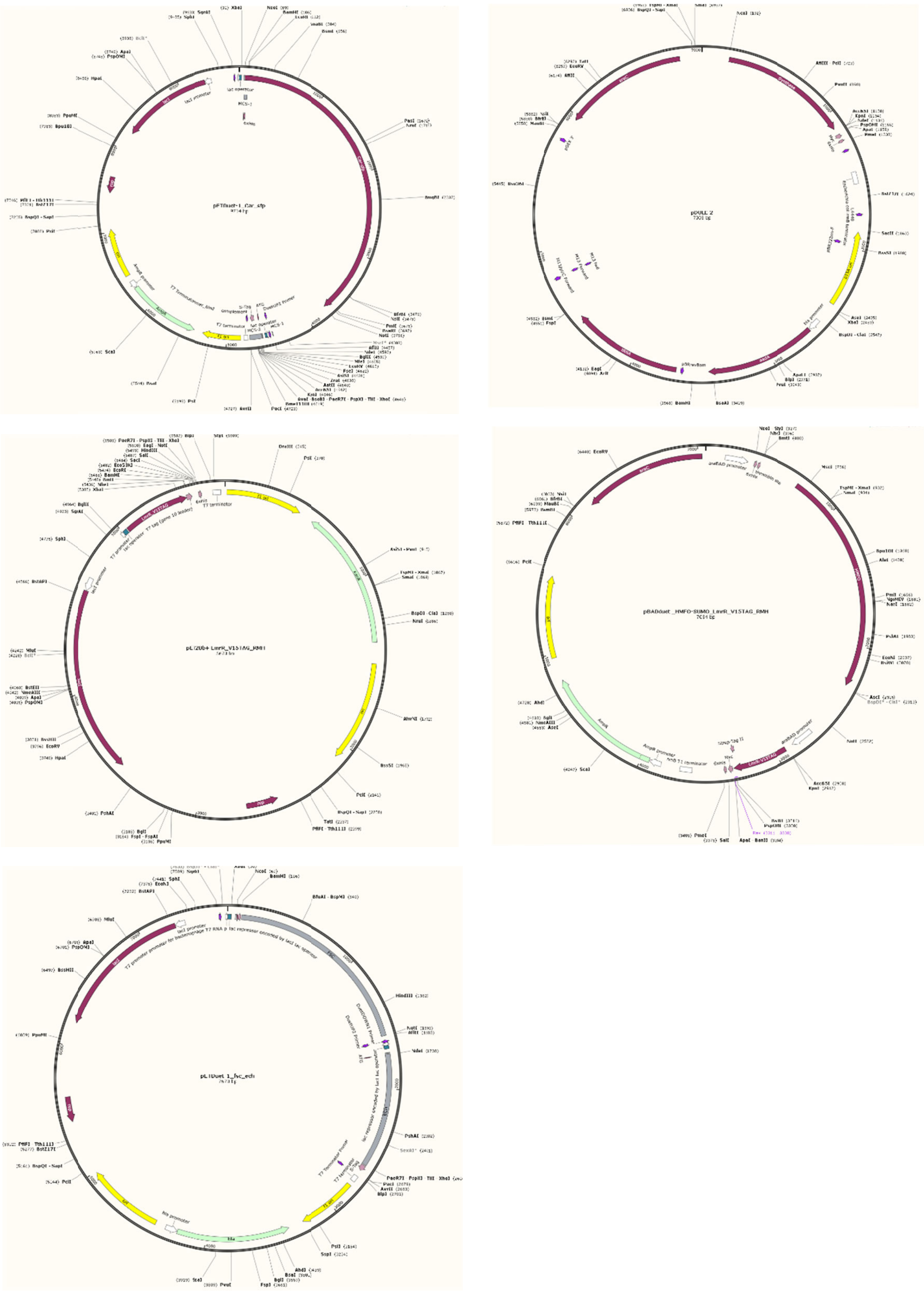

Figure S24. All pETDuet-1 vectors were ordered with the synthetic gene from genscript. The emplty vector of pET28b+ and pBADduet was received as a gift from Prof Marco Fraaije (University of Groningen).

Calibration Curves for Aldehydes
Benzaldehyde (1b) IS: 2-methylanisole

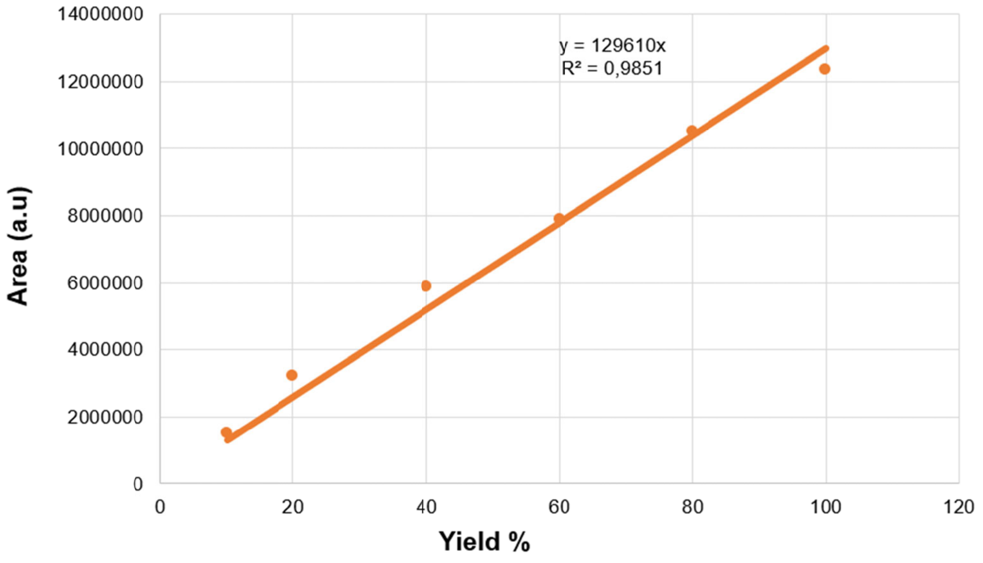

***p*-OH Benzaldehyde (1a)** IS: (2-Phenylquinoline)

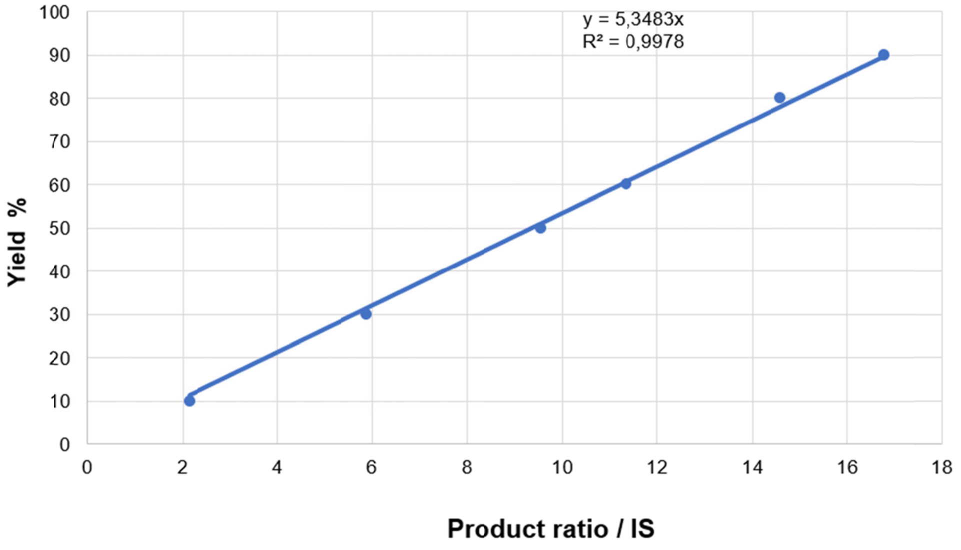

Chemical synthesis and calibration curves of hydrazone products 3a and 3b
The hydrazone product between NBD-H and 4-hydroxybenzaldehyde (4-HBA) was prepared according to the same procedure as we previously reported [5]'.The hydrazone product of the reaction between NBD-H and benzaldehyde was prepared

as previously described in the literature[6].[4] In brief, NBDH (25 mg, 0.13 mmol, 1 equiv) was dissolved in methanol (10 mL). Benzaldehyde (0.13 mL, 1.3 mmol, 10 equiv) was added, turning the reaction mixture a red color. The product gradually formed as a dark red-black precipitate and was isolated by vacuum filtration. 1H-NMR (400 MHz, DMSO-d6) 12.9 (m, 1H), 8.60 (m, 1H), 7.94−7.90 (m, 2H), 7.84 (m, 1H), 7.63−7.58 (m, 1H), 7.50-7.46 (m, 3H).

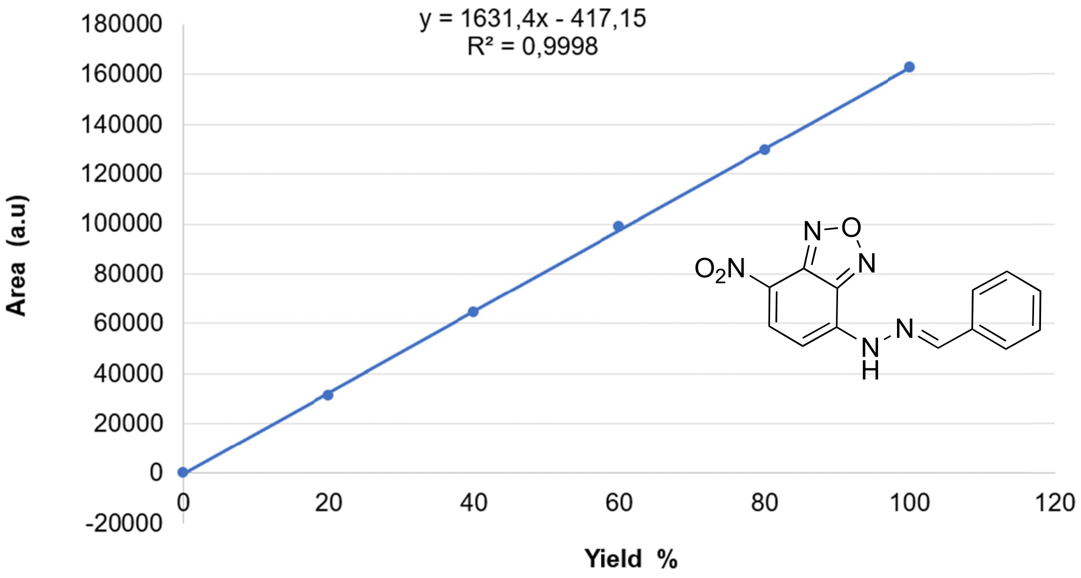

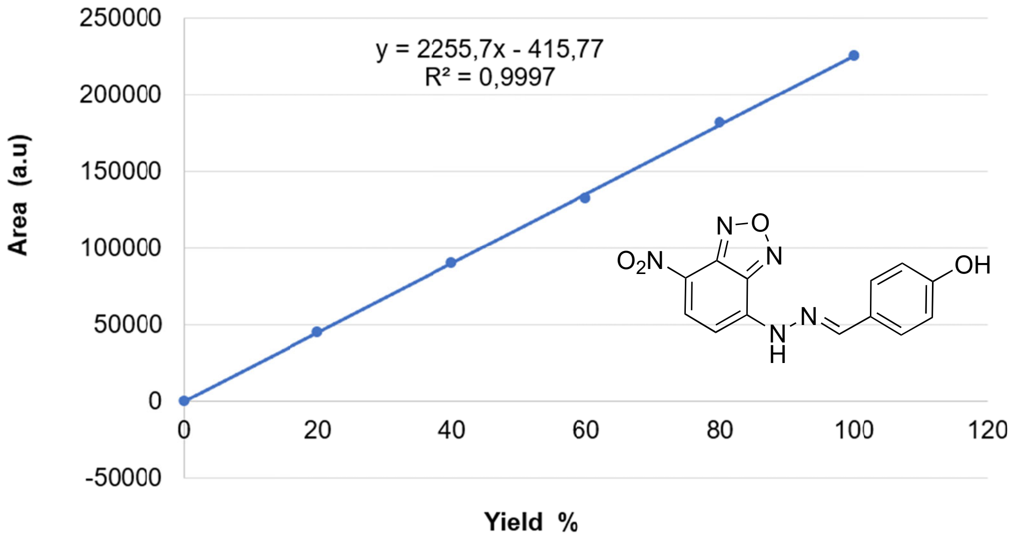

NMR spectra

1 H-NMR product **3b** in acetone-d6

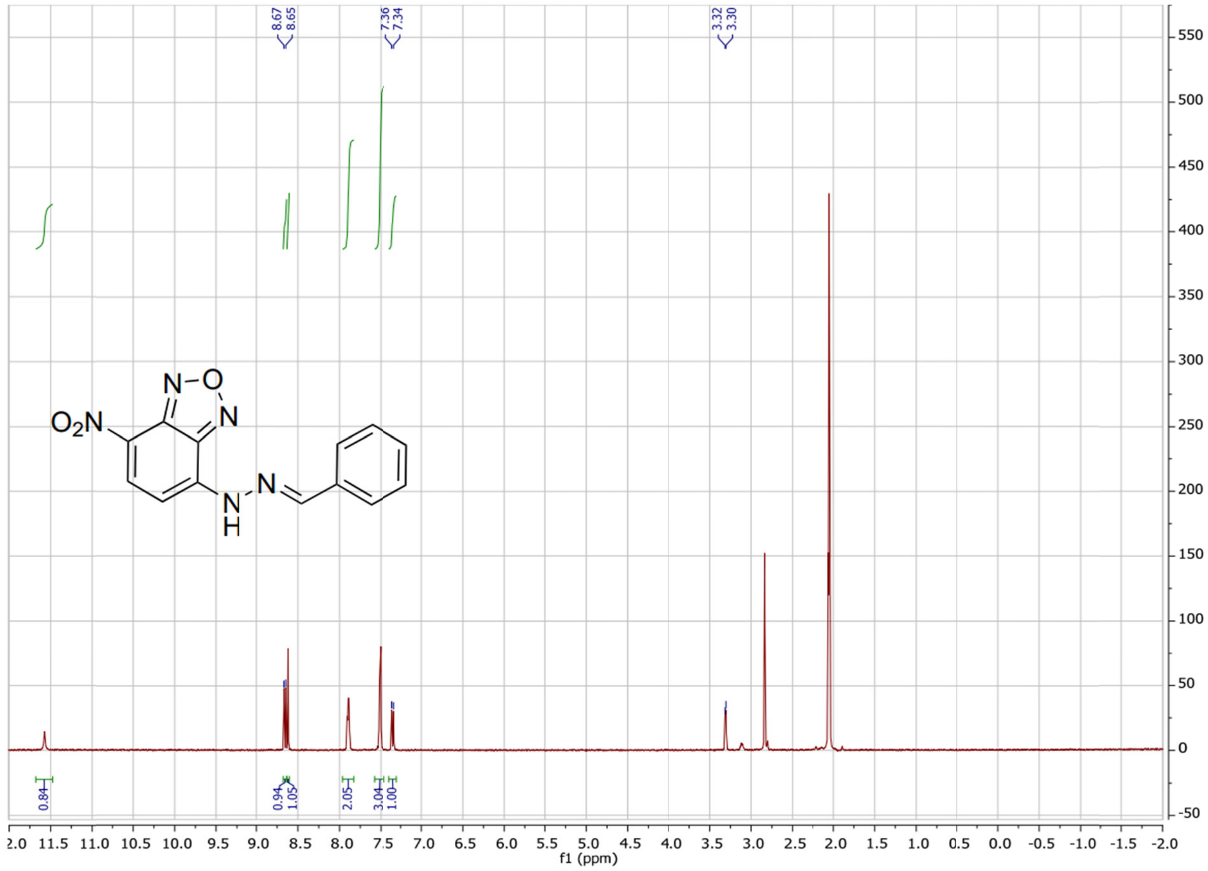

Scale up of in vivo hydrazone reaction using LmrR_V15pAF_RMH/HMFO system.

Experimental culture cells were grown in 2 L MMV supplemented with antibiotics at 37°C, 135 rpm until OD_{600} of 0.6. Gene expression was then induced with 0.02 % arabinose. 1 mM of pAF was also added in a solid form upon expression induction and incubated at 24 °C, agitated at 135 rpm for 48 hrs. Cultures were pelleted and resuspended in phosphate buffer at pH 6.5, to wash away non-incorporated UAA. In the 200 mL cell culture at OD_{600} of 25 in buffer, 26 mg of NBDH dissolved in 12 ml acetonitrile (ACN), 5 mM benzyl alcohol (final concentration) in acetonitrile were added and incubated at 24 °C / 135 rpm for overnight. Cells were then collected and spun down at 6000 rpm for 20 mins and collected the supernatant for product extraction. Pellet were then resuspend in 20 ml phosphate buffer and sonicated, the mixture was also spun down and collected the supernatant. The pellet was washed again 3x with 20 ml buffer and ACN (ratio 1:1). Sodium chloride (25 g) was added to the aqueous solution and the reaction mixture was extracted with EtOAc (3 x 300 mL). The remaining aqueous phase was then filtered through celite and extracted again with EtOAc (3 x 300 mL). The combined organic phases were dried with anhydrous Na_2SO_4 and all volatiles were removed to give the crude product. The product was purified via column chromatography (SiO_2, Pentane:EtOAc:MeOH=100:5:1). All fractions containing the product were collected giving the hydrazone in a mixture (total mass 8.9 mg).

NMR spectra 1H-NMR crude product **3b** in acetone-d6

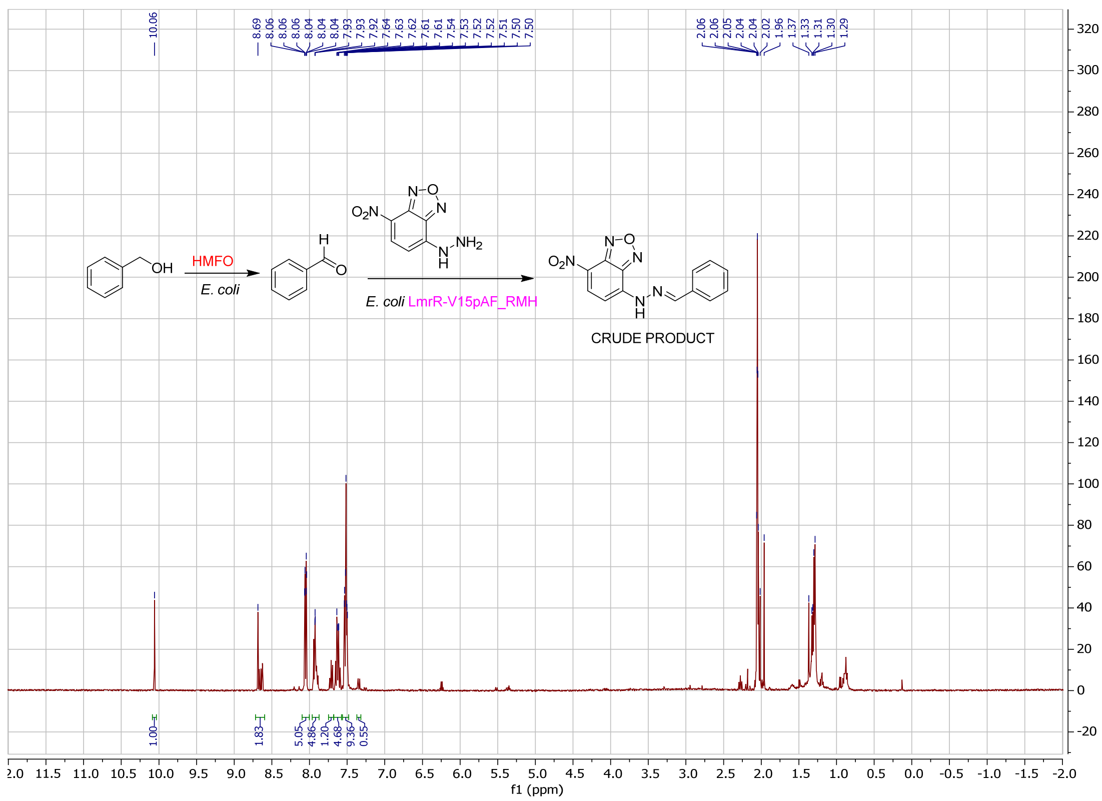

Crude product of **3b** after extraction

NMR spectra 1H-NMR product **3b** in acetone-d6

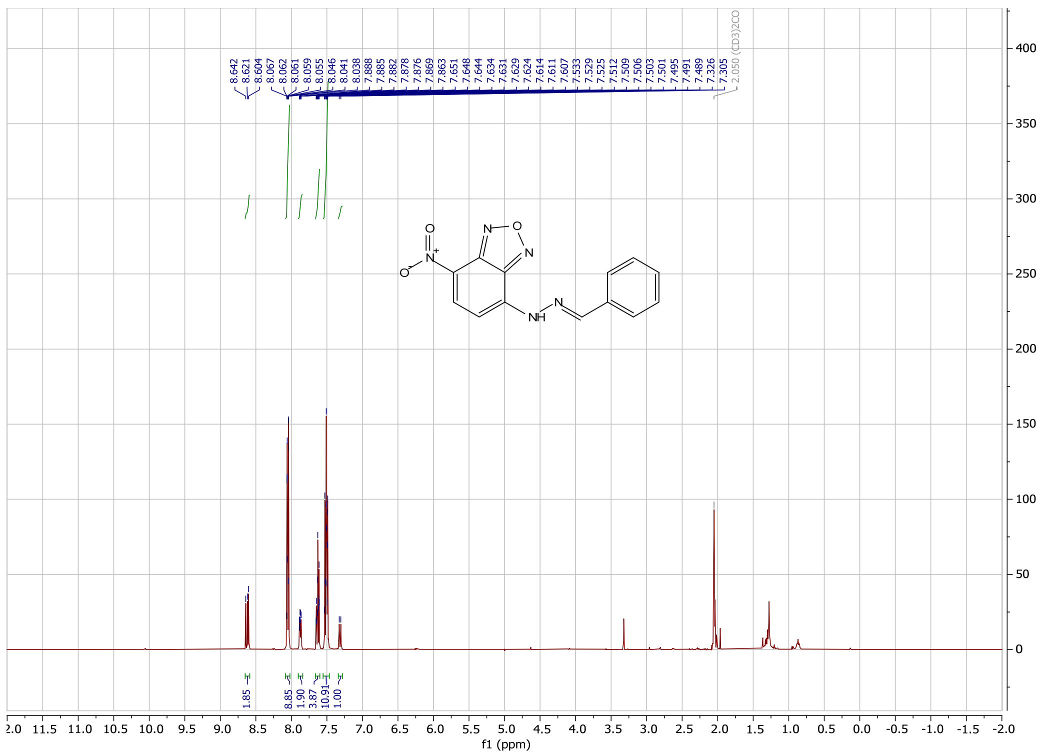

Product **3b** after column purification

The scale up of hydrazone formation using the LmrR_V15pAF_RMH/HMFO system was successful and the presence of benzaldehyde detected on the NMR shows the efficient oxidation of the benzyl alcohol by the HMFO. The product was extracted and purified by classical organic synthesis technique i.e. column chromatography. However, the yield was lower due to the substrate used (hydrazine), which makes it difficult to extract the product from the cell. As a prove of concept, this substrate was used for the reaction due to the chromogenic nature for easy screening.

References

[1] C. Mayer, C. Dulson, E. Reddem, A. M. W. H. Thunnissen, G. Roelfes, *Angew. Chemie - Int. Ed.* **2019**, *58*, 2083–2087.

[2] R. A. Mehl, J. C. Anderson, S. W. Santoro, L. Wang, A. B. Martin, D. S. King, D. M. Horn, P. G. Schultz, *J. Am. Chem. Soc.* **2003**, *125*, 935–939.

[3] E. M. Gabor, E. J. De Vries, D. B. Janssen, *Environ. Microbiol.* **2004**, *6*, 948–958.

[4] J. D.B., *Prog Ind Microbiol* **1984**, *29*, 169–178.

[5] I. Drienovská, C. Mayer, C. Dulson, G. Roelfes, *Nat. Chem.* **2018**, *10*, 946–952.

[6] J. A. Key, C. Li, C. W. Cairo, *Bioconjug. Chem.* **2012**, *23*, 363–371.
